# Methods for correcting inference based on outcomes predicted by machine learning

**DOI:** 10.1073/pnas.2001238117

**Published:** 2020-11-18

**Authors:** Siruo Wang, Tyler H. McCormick, Jeffrey T. Leek

**Affiliations:** ^a^Department of Biostatistics, Johns Hopkins Bloomberg School of Public Health, Baltimore, MD 21205;; ^b^Department of Statistics, University of Washington, Seattle, WA 98195;; ^c^Department of Sociology, University of Washington, Seattle, WA 98195

**Keywords:** statistics, machine learning, postprediction inference, interpretability

## Abstract

Many modern problems in medicine and public health leverage machine-learning methods to predict outcomes based on observable covariates. In a wide array of settings, predicted outcomes are used in subsequent statistical analysis, often without accounting for the distinction between observed and predicted outcomes. We call inference with predicted outcomes postprediction inference. In this paper, we develop methods for correcting statistical inference using outcomes predicted with arbitrarily complicated machine-learning models including random forests and deep neural nets. Rather than trying to derive the correction from first principles for each machine-learning algorithm, we observe that there is typically a low-dimensional and easily modeled representation of the relationship between the observed and predicted outcomes. We build an approach for postprediction inference that naturally fits into the standard machine-learning framework where the data are divided into training, testing, and validation sets. We train the prediction model in the training set, estimate the relationship between the observed and predicted outcomes in the testing set, and use that relationship to correct subsequent inference in the validation set. We show our postprediction inference (postpi) approach can correct bias and improve variance estimation and subsequent statistical inference with predicted outcomes. To show the broad range of applicability of our approach, we show postpi can improve inference in two distinct fields: modeling predicted phenotypes in repurposed gene expression data and modeling predicted causes of death in verbal autopsy data. Our method is available through an open-source R package: https://github.com/leekgroup/postpi.

## Significance

Machine learning is now being used across the entire scientific enterprise. Researchers commonly use the predictions from random forests or deep neural networks in downstream statistical analysis as if they were observed data. We show that this approach can lead to extreme bias and uncontrolled variance in downstream statistical models. We propose a statistical adjustment to correct biased inference in regression models using predicted outcomes—regardless of the machine-learning model used to make those predictions.

The past decade has seen an explosion both in data available for precision health (1–3) and, simultaneously, in user-friendly tools such as the caret package (4) and Scikit-learn (5) that make implementing complex statistical and machine-learning methods possible for an increasingly wide range of scientists. For example, machine learning from electronic medical records is used to predict phenotypes (6, 7), genomic data are used to predict health outcomes (8), and survey data are used to predict the cause of death in settings where deaths happen outside of hospitals (9, 10). The increased focus on ideas like precision medicine means the role of machine learning in medicine and public health will only increase (11). As machine learning plays an increasingly critical role across scientific disciplines, it is critical to consider all sources of potential variability in downstream inference to ensure stable statistical results (12, 13).

In many settings, researchers do not observe outcomes directly, so observed outcomes are often replaced with predicted outcomes from machine-learning models in downstream analyses (6, 14–18). One example from genetics is association studies between genetic variants and Alzheimer’s disease for young adults. Because young adults have not developed Alzheimer’s disease, it is difficult to associate the phenotype with genetic variants. However, these adults’ older relatives can be used to predict the ultimate phenotype of participants in the study using known inheritance patterns for the disease. The predicted outcome can be used in place of the observed Alzheimer’s status when performing a genome-wide association study (15).

This is just one example of the phenomenon of postprediction inference (postpi). Although common, this approach poses multiple statistical challenges. The predicted outcomes may be biased, or the predicted outcomes may have less variability than the actual outcomes. Standard practice in many applications is to treat predicted outcomes as if they were observed outcomes in subsequent regression models (6, 14–18). As we will show, uncorrected postprediction inference will frequently have deflated standard errors, bias, and inflated false positive rates.

Postprediction inference appears across fields and has been recognized as a potential source of error in recent work on prevalence estimation (see for example refs. 19 and 20 in the context of dataset shift and ref. 21 in document class prevalence estimation). Here, we focus on developing analytical and bootstrap-based approaches to correct regression estimates, SEs, and test statistics in inferential regression models using predicted outcomes. We examine settings where a predicted outcome becomes the dependent variable in the subsequent inferential regression analysis. We derive an analytical correction in the case of linear regression and bootstrap-based corrections for more general regression models, focusing on linear and logistic regression as they are the most common inferential models. Our bootstrap-based approach can, however, easily be extended to any generalized linear regression inference model.

**Fig. 1.**
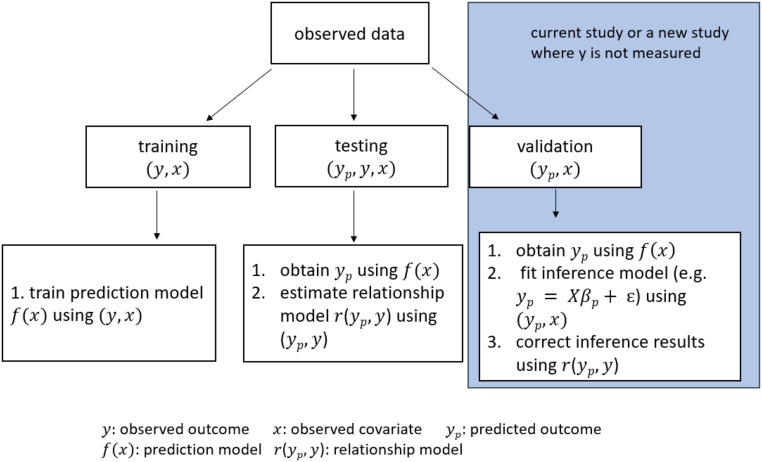
Data split diagram. The common structure of our approach is to divide the observed data into training, testing, and validation sets. The training set is used to train the prediction model, the testing set is used to estimate the relationship between observed and predicted outcomes, and the validation set is for fitting the downstream inferential model where the relationship model is used to correct inference in the subsequent statistical inference.

Both our analytical and bootstrap-based corrections take advantage of the standard structure for machine-learning problems. We assume that we have at least three separate subsamples, which we here label as training set, testing set, and validation set (Fig. 1). We assume that the data-generating distribution for the three datasets is the same and that the training and testing sets are complete—we observe both the outcome of interest (y) and the covariates (x). In the validation set, we assume that only the covariates are observed. The validation set could represent either a validation subset from a single sample or a future prospectively collected dataset where we wish to perform inference but it is too costly or challenging to collect the outcome.

A prediction function for the outcome ($y_{p}=\hat{f}\left(x\right)$) is generated in the training set and applied in the testing and validation sets. In the validation set our goal is to perform inference on a regression model of the form g(E[y|X])=Xβ. However, in the validation set, only the covariates are observed so instead we must fit $g\left(E\left[y_{p}|X\right]\right)=X\beta_{p}$. Our goal is to recover the inference we would have obtained if we had observed the true outcomes y in the validation set. To correct inference using the predicted outcomes, we take advantage of the testing dataset where we have both the predicted ($y_{p}$) and observed (y) outcomes. We derive a correction for inference using $y_{p}$ based on the relationship between y and $y_{p}$.

An advantage of this approach is that it is not specific to a particular machine-learning model. That is, we do not need to know a priori the expected out-of-sample operating characteristics for a given method. Instead, we assume that the relationship between the predicted and observed outcomes in the testing set well characterizes the same relationship in the validation set.

The setting we describe has parallels with multiple imputation (22) for missing data, but has several distinct features. Any prediction problem could be cast as a missing data problem where all of the values are missing and no missingness mechanism distinguishes observed and unobserved outcomes. The reason is that in the validation set or subsequent analyses in practical problems, there are no observed outcome data. Multiple imputation also frequently relies on a generative model for simulating data. However, in our setting, we wish to build a framework that can be used for any machine-learning model, regardless of its operating characteristics. We, therefore, need a methodology that can use a black-box machine-learning algorithm, but build a simple model for the relationship between predicted and observed outcome data. This problem is also related to the idea of errors in variables (23) or measurement error models (24), where either the outcome or the covariates are measured with error. However, in prediction problems, we can no longer assume that the errors are independent of the predicted values, since the machine-learning predictions may be more accurate for subsets of the y values.

Aside from its utility in medicine and public health, the method we propose is also broadly applicable in the social sciences. In political science, for example, researchers use machine-learning tools to classify sentiment or political identification in segments of text and then fit regression models to identify features of text leaning toward one party or another (25). In sociology, researchers use machine-learning tools to infer the race of household heads subject to eviction and then use regression models to understand heterogeneity in circumstances related to evictions of individuals of a particular race (26).

Here, we apply our postpi approach to two open problems: modeling the relationship between gene expression levels and tissue types (8) and understanding trends in (predicted) cause of death (27, 28). We show that our method can reduce bias, appropriately model variability, and correct hypothesis testing in the case where only the predicted outcomes are observed. We also discuss the sensitivity of our approach to changes in the study population that might lead to a violation of the assumptions of our approach. Our postpi approach is available as an open-source R package available from GitHub: https://github.com/leekgroup/postpi.

## Illustrative Example

We begin with an illustrative simulated example to highlight the issues that can arise with uncorrected postprediction inference. The methods we present in the subsequent sections cover a wider range of settings and do not require the distributional assumptions we make here for exposition. Here we simulate observations for the outcome $y_{i}$ and covariates $x_{ij}$ for i=1,…,n,j=1,…,p. We use $x_{i}$ to denote vector $\left[x_{i1},\ldots ,x_{ip}\right]$. In our simulation, we generate data according to the following true relationship between y and x which we denote by f(⋅):

$$y_{i}=f\left(x_{i}\right)+e_{u_{i}}.$$ [1]

This model represents the true underlying data-generating distribution, which is unknown in actual analysis settings.

Linear or generalized linear models are common approaches to perform inference, even when the data-generating process is unknown. We use $X_{i}$ to denote the design matrix. For example, we may be interested in fitting models of the form

$$y_{i}=X_{i}\beta +e_{i_{i}}.$$ [2]

We assume that we are in the setting where the outcome $y_{i}$ is too expensive or time consuming to collect. Instead, we use a prediction model of the form

$$y_{pi}=\hat{f}\left(x_{i}\right)$$ [3]

to predict the outcome. The prediction model may be arbitrarily complicated since the goal of the prediction is to minimize a suitable loss function, E‖y−f(x)‖, not to perform inference on the relationship between y and x.

Then, we fit the regression model of interest, using the predicted outcomes $y_{pi}$:

$$y_{pi}=X_{i}\beta_{p}+e_{p_{i}}.$$ [4]

Since the observed outcomes $y_{i}$ are not available, we instead use the predicted $y_{pi}$ to get a coefficient estimate ${\hat{\beta }}_{p}$ based on the model fit using $y_{pi}$ as the outcome, such that $E\left(y_{pi}|X_{i}\right)=X_{i}\beta_{p}$. Eq. **4** no long appropriately reflects our uncertainty about the outcome—leading to bias in the estimates, SEs that are too small, and anticonservatively biased *P* values and false positives.

**Fig. 2.**
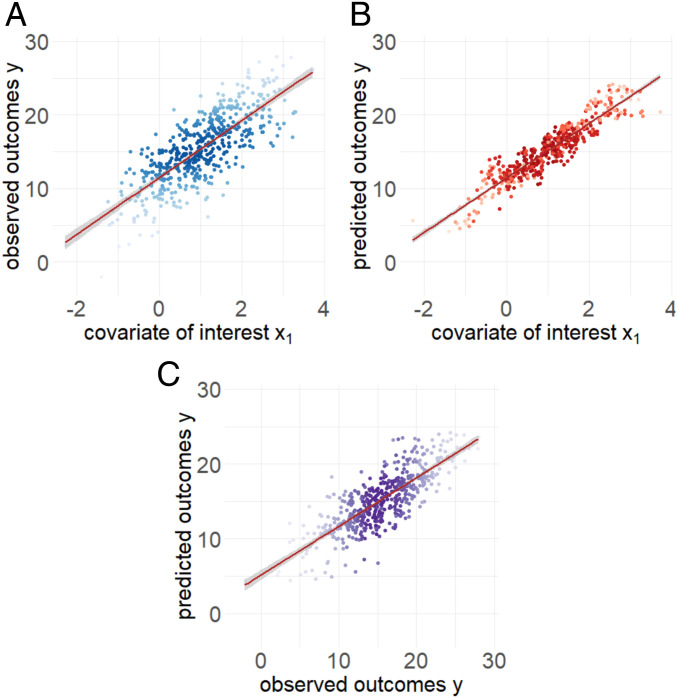
Simulated example. Data were simulated from the ground truth model as a linear model. (*A*) Observed outcomes versus the covariate of interest. The *x* axis shows the covariate of interest $x_{1}$ and the *y* axis shows the observed outcomes of y. (*B*) Predicted outcomes versus the covariate of interest. The *x* axis shows the covariate of interest $x_{1}$ and the *y* axis shows the predicted outcomes of $y_{p}$. (*C*) Observed outcomes versus predicted outcomes. The *x* axis shows the observed outcomes of y and the *y* axis shows the predicted outcomes of $y_{p}$.

Fig. 2 shows the results of our simulated example. We simulate covariates $x_{i1}$, $x_{i2}$, $x_{i3}$, and $x_{i4}$ and error terms $e_{u_{i}}$ from normal distributions and simulate the observed outcome $y_{i}$ using a simple regression model as the state of nature, f(⋅). Then we separate the simulated values into training, testing, and validation sets that have the same data-generating systems, and we follow the same procedure in each set as described in the data split diagram in Fig. 1. In the training set, we train a random forest (29, 30) model as the prediction model $\hat{f}\left(\cdot \right)$ using all covariates $x_{i1}$, $x_{i2}$, $x_{i3}$, and $x_{i4}$ and observed outcome $y_{i}$. In the testing set, we apply this prediction model to the observed covariates $x_{i1}$, $x_{i2}$, $x_{i3}$, and $x_{i4}$ to obtain the predicted values $y_{pi}=\hat{f}\left(x_{i}\right)$. Then we estimate the relationship between the predicted and observed outcomes. In the validation set, we fit a linear regression model as the inference model.

This simulation is designed to highlight the issues that arise with postprediction inference in a setting where both $y_{i}$ and $y_{pi}$ are available. In actual data analysis with predicted outcomes, we would not observe the true $y_{i}$ in the validation set and all inference would be performed with $y_{pi}$.

In Fig. 2*A* we illustrate the true relationship between the simulated y and $x_{1}$ (blue color). In Fig. 2*B* we show the predicted values $y_{p}$ versus $x_{1}$ (red color). In Fig. 2*B*, the relationship has changed, with different slope and variance. In Fig. 2*C* we show the relationship between the observed and predicted outcomes. In this simulated example, we know that the estimated coefficient for the relationship between the observed outcome y and x is 3.87 with a SE of 0.14. However, when we fit the model using the predicted outcome $y_{p}$, we get an estimate of 3.7 with a SE of 0.068. This simple simulated example illustrates that inferences drawn with predicted outcomes may have 1) biased estimates, 2) too small SEs, and hence 3) *P* values and inference that are anticonservatively biased.

To adjust for error in predictions, one option would be to derive bias and SE corrections for a specific machine-learning method. This approach would leverage knowledge about how a specific prediction tool works. To compute the bias and SEs analytically, we both 1) need to know what machine-learning model was used and 2) need to be able to theoretically characterize the properties of that machine-learning model’s predictions. This approach would restrict an analyst to only machine-learning approaches whose inferential operating characteristics have been derived. Fig. 2*C* suggests an alternative approach. In this case, the relationship between the observed and predicted outcome can easily be modeled using linear regression. We will show that this observation holds for a variety of machine-learning techniques.

The key idea of our approach is that we use the relationship between the predicted and observed data in the testing set, to estimate the bias and variance introduced by using predicted outcome as the dependent variable in the downstream inferential regression model in the validation set. This approach does not require idiosyncratic information about each machine-learning approach and, instead, assumes that a relatively simple model captures the relationship between the predicted and observed outcomes.

## Method

### Overview of Our Approach.

Our goal is to develop a method for correcting inference for parameters in an inferential regression model where predicted outcomes are treated as observed outcomes.

We make the following assumptions about the structure of the data and model. We assume that the data are generated from an unknown data-generating model of the form

$$g\left[E\left(y_{i}|x_{i1},x_{i2},\ldots ,x_{ip}\right)\right]=f\left(x_{i1},x_{i2},\ldots ,x_{ip}\right).$$ [5]

This model represents the “true state of nature” but is not directly observed in any practical problem.

We also assume that in a new dataset, it may be too expensive, too time consuming, or too difficult to collect outcome variable $y_{i}$ for all samples. We, therefore, attempt to predict this outcome with an arbitrary machine-learning algorithm $\hat{f}\left(\cdot \right)$ so that $y_{pi}=\hat{f}\left(x_{i1},x_{i2},\ldots ,x_{ip}\right)$ is the predicted outcome based on the observed covariates. However, the primary goal of our analysis is not to simply predict outcomes but to perform inference in the new dataset on the relationship between the outcomes and the covariates. This must be a subset of covariates used in the prediction model $\hat{f}\left(\cdot \right)$ (see *SI Appendix*, section 2C for further discussion of this assumption).

In practice, the true data-generating process is rarely known. The common statistical practice is to fit linear or generalized linear models to relate outcomes to covariates for inference (6, 14–18). Letting $X_{i}$ denote a covariate of interest in matrix notation, then a typical regression model may be of the form

$$g\left[E\left(y_{i}|X_{i}\right)\right]=X_{i}\beta .$$ [6]

When the outcome is observed, we can directly compute the estimate of β. However, here we consider the case where it will not be possible to observe the outcome in future datasets due to cost or inconvenience, so the predicted outcome $y_{pi}$ will be used in Eq. **6**.

The most direct approach to performing postprediction inference is to use predicted outcomes and ignore the fact that they are predicted. However, this approach can lead to bias in the estimates, small SEs, anticonservative test statistics, and false positives for estimated coefficients as we saw in the simple example in *Illustrative Example*. We will demonstrate that this approach produces consistently inaccurate inference in the simulation and real application settings. Despite these potential biases, this approach to direct use of predicted outcomes in inferential models is popular in genomics (18), genetic (15), public health (10), and electronic health record phenotyping (6) among other applications.

Another strategy would be to try to directly derive the properties of the coefficients and SEs in the subsequent inference model using the definition of the machine-learning algorithm $\hat{f}\left(\cdot \right)$. When a prediction is based on a sufficiently simple machine-learning algorithm, this may be possible to do directly. However, machine-learning models now commonly include complicated algorithmic approaches involving thousands or millions of parameters, including k-nearest neighbors (31), support vector machine (SVM) (32), random forest (29, 30), and deep neural network (33).

**Fig. 3.**
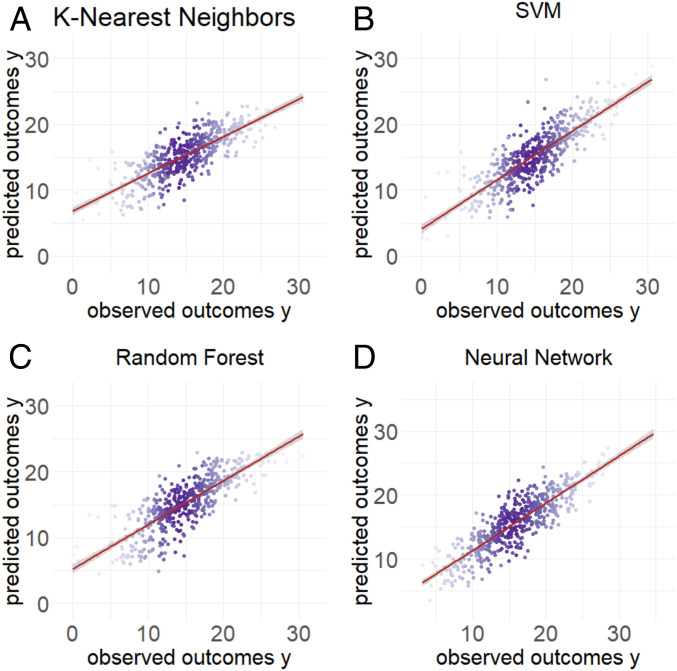
Relationship between the observed and predicted outcomes using different machine-learning models. Data were simulated from the ground truth model as a linear model with normally distributed noise. On the *x* axis is the observed outcome of y and on the *y* axis are the predicted outcomes $y_{p}$. We show that regardless of the prediction method, (*A*) k-nearest neighbors, (*B*) random forest, (*C*) SVM, or (*D*) neural network, the observed and predicted outcomes follow a distribution that can be accurately approximated with a linear regression model.

We instead focus on modeling the relationship between the observed and predicted outcomes. Our key insight is that even when we use a complicated machine-learning tool to predict outcomes, a relatively simple model can describe the relationship between the observed and predicted outcomes (Fig. 3). We then use this estimated relationship to compute bias and SE corrections for the subsequent inferential analyses using predicted values as the dependent variable.

Based on the observation in Fig. 3, we relate the observed to the predicted data through a flexible model k(⋅):

$$y_{pi}=k\left(y_{i}\right).$$ [7]

For continuous outcomes, we can estimate this relationship as a linear regression model. For categorical outcomes, we can use a logistic regression model or a simple machine-learning model. To fit this relationship model, we take advantage of the standard structure of machine-learning model development. In these problems, the observed data are split into training, testing, and validation sets, and we assume that the three sets have the same data-generating system. As illustrated in Fig. 1, we can build a prediction model in the training set and then compute an unbiased estimate of the relationship model in the testing set. Using this relationship model, we derive a correction for the estimates, SEs, and test statistics for our inference model. Then in the validation set, we can evaluate the quality of our correction in an independent sample.

In the following two sections, we derive bootstrap-based and analytical methods to correct inference for parameters in an inferential model on future datasets where predicted outcomes are treated as observed outcomes. For both methods, we generalize the approach to split the data into training, testing, and validation sets, and we assume that the three sets follow the same data-generating procedures. With either method, we assume that the covariates of interest in the subsequent inferential model must be a subset of covariates observed in the training and testing sets and used in the prediction model $\hat{f}\left(\cdot \right)$. Our methods do not provide the optimal inference correction results in the case where new covariates (not observed in the training and testing sets) are introduced as independent variables in the downstream inferential model (see *SI Appendix*, section 2C for an example and figures).

In *Bootstrap-Based Correction*, we develop a flexible bootstrap procedure for postprediction inference correction. The bootstrap-based approach allows for flexibility in both the relationship model and the subsequent inferential models. This approach is applicable provided that the relationship can be modeled through any sufficiently simple relationship that allows bootstrap sampling. In *Analytical Correction*, we derive an analytic correction that can be applied subject to additional assumptions. For the analytical derivation, we assume that 1) the outcome is continuous in the training, testing, and validation sets, 2) the relationship between the observed and predicted outcomes can be modeled using a normal linear regression model, and 3) the inferential goal is a linear regression model in the validation set. Under these assumptions, the analytic correction holds regardless of the choice of machine-learning algorithm used to make the predictions.

### Bootstrap-Based Correction.

In this section, we propose a bootstrap-based approach for correcting the bias and variance in the downstream inferential analyses. This approach can be applied for continuous, nonnormal data, categorical data, or count data. For our approach we make the following assumptions: 1) We have a training set to build the prediction model, a testing set to estimate parameters of the relationship model, and a validation set to fit a generalized regression model as the subsequent inferential model, and all three sets must follow the same data-generating system; 2) the relationship between the observed and predicted outcomes can be modeled through a flexible but specific simple model in the form of $y_{i}=k\left(y_{pi}\right)$ that is easy to sample from; 3) the relationship model will hold in future samples; and 4) the covariates of interest in the subsequent inferential analyses must be variables already seen in the training and testing sets and used in the prediction model.

The first step of our bootstrap procedure is to randomly split the data into training, testing, and validation sets, as illustrated in Fig. 1. The algorithm then proceeds as follows:

#### Bootstrap procedure.

1) Use the observed outcomes and covariates in the training set $\left(y_{\left(tr\right)},x_{\left(tr\right)}\right)$ to estimate a prediction model $y_{p}=\hat{f}\left(x\right)$.
2) Use the observed outcomes and predicted outcomes in the testing set $\left(y_{\left(te\right)},y_{p\left(te\right)}\right)$ to estimate the relationship model $y=k\left(y_{p}\right)$, where k(⋅) can be any flexible function.
3) Use the predicted outcomes and observed covariates in the validation set $\left(y_{p\left(val\right)},x_{\left(val\right)}\right)$ to bootstrap as follows:

##### Bootstrap iteration b = 1 to B.

i) For i=1,2,…,n, sample predicted values and the matching covariates $\left(y_{pi\left(val\right)}^{b},x_{i\left(val\right)}^{b}\right)$ with replacement.
ii) Simulate values from the relationship model ${\tilde{y}}_{i}^{b}=k\left(y_{pi\left(val\right)}^{b}\right)$ using the function k(⋅) estimated from the testing set in step 2.
iii) Fit the inference model $g\left[E\left({\tilde{y}}^{b}|X_{\left(val\right)}^{b}\right)\right]=X_{\left(val\right)}^{b}\beta^{b}$ using the simulated outcomes ${\tilde{y}}^{b}$ which build in the prediction error from the relationship model and the matching model matrix based on the sampled covariates in matrix notation $X^{b}$.
iv) Extract the coefficient estimator ${\hat{\beta }}^{b}$ from the fitted inference model in iii.
v) Extract the SE of the estimator $se\left({\hat{\beta }}^{b}\right)$ from the fitted inference model in iii.
4) Estimate the inference model coefficient using a median function on the estimators ${\hat{\beta }}^{b}$ collected in step 3, iv: ${\hat{\beta }}^{boot}=median\left({\hat{\beta }}^{1},{\hat{\beta }}^{2},\ldots ,{\hat{\beta }}^{B}\right)$.
5) Estimate the inference model SE:
a) For the parametric method, use a median function on the SEs $SE\left({\hat{\beta }}^{b}\right)$ collected in step 3, v: ${\hat{SE}}^{boot,par}=median\left(\hat{SE}\left({\hat{\beta }}^{1}\right),\hat{SE}\left({\hat{\beta }}^{2}\right),\ldots ,\hat{SE}\left({\hat{\beta }}^{B}\right)\right)$.
b) For the nonparametric method, use the SE of the estimators ${\hat{\beta }}^{b}$ collected in step 3, iv: ${\hat{SE}}^{boot,non-par}=SD\left({\hat{\beta }}^{1},{\hat{\beta }}^{2},\ldots ,{\hat{\beta }}^{B}\right)$.

The bootstrap-based approach builds in two types of errors: the error due to random sampling and the prediction error. The prediction error is introduced by sampling from the relationship model in the for loop, step 3, ii. We again make the simplifying assumption that y and $y_{p}$ can be related through a model that is easy to fit. We can focus here on the class of generalized linear models, but in the Bootstrap Procedure step 2, the relationship function k(⋅) could be more general, even flexible as a machine-learning algorithm, provided it can be easily estimated and sampled. The advantage of the relationship model is that we do not need to assume the type or complexity of the prediction function $\hat{f}\left(\cdot \right)$. It can be arbitrarily complicated as long as the estimated relationship between the observed and predicted values can be sampled.

### Analytical Correction.

In this section, we propose an analytical method to correct inferences for the parameters in the downstream linear model. We assume that the data have been divided into training (tr), testing (te), and validation sets (val) and that the data-generating distribution is the same across the three sets: $y∼N\left(f\left(x\right),\sigma_{t}^{2}\right)$, where f(⋅) is an arbitrary and unknown function of the covariates. In the training set, we use the observed outcomes and covariates $\left(y_{\left(tr\right)},x_{\left(tr\right)}\right)$ to estimate a prediction model $y_{p}=\hat{f}\left(x\right)$. In the testing set, we use the predicted and observed outcomes $\left(y_{\left(te\right)},y_{p\left(te\right)}\right)$ to estimate a linear relationship model. In the validation set, we would fit a linear inference model using predicted outcomes and covariates in matrix notation $\left(y_{p\left(val\right)},X_{\left(val\right)}\right)$. Our goal is to infer the relationship between the outcome y and some subset of the covariates in the validation set or a future dataset where a collection of outcomes is either prohibitively expensive or complicated.

The analytical derivation approach computes the corrected parameters in the inference model more efficiently than the bootstrap-based approach, but with more restrictions in the assumptions to calculate a closed-form solution to the parameters in the downstream inferential model: 1) We concentrate on a setting where the outcome can only be continuous and approximately normally distributed, 2) the relationship model estimated in the testing set is also approximately normally distributed, and 3) the subsequent inferential model must be a linear model that we can correct inference from.

In the validation set, ideally we would fit the model

$$y_{\left(val\right)}|X_{\left(val\right)}∼N\left(X_{\left(val\right)}\beta_{\left(val\right)},\sigma_{i}^{2}\right).$$ [8]

However, the outcome is not observed in the validation set. Instead, we fit the model

$$y_{p\left(val\right)}|X_{\left(val\right)}∼N\left(X_{\left(val\right)}\beta_{p\left(val\right)},\sigma_{p}^{2}\right).$$ [9]

In this case, we are no longer estimating the same quantity due to the change in the dependent variable. This uncorrected strategy to postprediction inference is commonly used in real practice (6, 14–18). Our goal here is to develop a correction to recover the inference about $\beta_{\left(val\right)}$ as if the observed outcomes were available.

We can use information about the relationship between the observed and predicted outcomes to correct inference in datasets where y is not observed and we substitute $y_{p}$. We assume a relationship model

$$y_{\left(te\right)}∼N\left(\gamma_{0}+\gamma_{1}y_{p\left(te\right)},\sigma_{r}^{2}\right).$$ [10]

The key observation we have made is that a simplified model often holds, even when the machine-learning function used to make the predictions $\hat{f}\left(x\right)$ is quite complicated (Fig. 3).

Our goal is not to model the full distribution of ($y_{\left(val\right)}$, $y_{p\left(val\right)}$, $x_{\left(val\right)}$), but instead to infer the relationship between the outcome $y_{\left(val\right)}$ and a set of covariates $x_{\left(val\right)}$. If we had observed $y_{\left(val\right)}$ and fit the inference model as shown in Eq. **8**, we have the ordinary least-squares estimator ${\hat{\beta }}_{\left(val\right)}={\left(X_{\left(val\right)}^{T}X_{\left(val\right)}\right)}^{-1}X_{\left(val\right)}^{T}y_{\left(val\right)}$. However, $y_{\left(val\right)}$ is unobserved and thus ${\hat{\beta }}_{\left(val\right)}$ cannot be calculated directly. So, we first want to estimate $y_{\left(val\right)}$ using the conditional expectation $E\left[y_{\left(val\right)}|X_{\left(val\right)}\right]$. This expectation can be written as

$$\begin{array}{cc} E\left[y_{\left(val\right)}|X_{\left(val\right)}\right] & =E\left[E\left(y_{\left(val\right)}|X_{\left(val\right)},y_{p\left(val\right)}\right)|X_{\left(val\right)}\right]\\ & \approx E\left[E\left(y_{\left(val\right)}|y_{p\left(val\right)}\right)|X_{\left(val\right)}\right]\\ & =\gamma_{0\left(te\right)}+\gamma_{1\left(te\right)}\;X_{\left(val\right)}\beta_{p\left(val\right)}. \end{array}$$ [11]

Here $\beta_{p\left(val\right)}$ represents the parameter in the linear regression inference model where predicted outcome is used as the dependent variable. The approximation in Eq. **11** is based on using the relationship between the predicted outcome and observed outcome $E\left(y_{\left(val\right)}|y_{p\left(val\right)}\right)$ as an approximation to the conditional expectation $E\left(y_{\left(val\right)}|X_{\left(val\right)},y_{p\left(val\right)}\right)$ (see *SI Appendix*, section 1A.1 for full analytical derivation).

This approximation can be made exactly in the extreme scenario where the predicted outcome exactly captures the relationship between the outcome and the covariates $y_{p}=f\left(x\right)$. In this case, the real outcome can be written as $y=y_{p}+\epsilon$, and we have exactly $E\left(y_{\left(val\right)}|X_{\left(val\right)},y_{p\left(val\right)}\right)=E\left(y_{\left(val\right)}|y_{p\left(val\right)}\right)$ (see *SI Appendix*, section 1A.4 for a full analytical derivation). Thus, we can approximate the unobserved outcome $y_{\left(val\right)}$ as

$$y_{\left(val\right)}^{⋆}={\hat{\gamma }}_{0\left(te\right)}+{\hat{\gamma }}_{1\left(te\right)}X_{\left(val\right)}{\hat{\beta }}_{p\left(val\right)}$$ [12]

and we then approximate the estimator ${\hat{\beta }}_{\left(val\right)}$ as

$${\hat{\beta }}_{\left(val\right)}^{⋆}={\left(X_{\left(val\right)}^{T}X_{\left(val\right)}\right)}^{-1}X_{\left(val\right)}^{T}\left({\hat{\gamma }}_{0\left(te\right)}+{\hat{\gamma }}_{1\left(te\right)}X_{\left(val\right)}{\hat{\beta }}_{p\left(val\right)}\right).$$ [13]

Through this approximation, we further show that $E\left({\hat{\beta }}_{\left(val\right)}^{⋆}|X_{\left(val\right)}\right)\approx \beta_{\left(val\right)}$ (see *SI Appendix*, section 1A.2 for full analytical derivation).

To make inferences, we also need to estimate the SE of the estimator ${\hat{\beta }}_{\left(val\right)}$. The challenge is that the SE cannot be simply calculated by fitting the regression model in Eq. **8** because $y_{\left(val\right)}$ is unobserved. Instead, we first estimate the conditional variance $Var\left[y_{\left(val\right)}|X_{\left(val\right)}\right]$ using the variance that comes from both the relationship model in Eq. **10** and the inference model in Eq. **9** with predicted outcomes. This is a similar approach to the expectation derivation above where we assume that the observed outcome is unknown. Using the law of total conditional variance

$$\begin{array}{cc} Var & \left[y_{\left(val\right)}|X_{\left(val\right)}\right]\\ & =E\left[Var\left(y_{\left(val\right)}|y_{p\left(val\right)},X_{\left(val\right)}\right)|X_{\left(val\right)}\right]\\ & \;+Var\left[E\left(y_{\left(val\right)}|y_{p\left(val\right)},X_{\left(val\right)}\right)|X_{\left(val\right)}\right]\\ & \approx E\left[Var\left(y_{\left(val\right)}|y_{p\left(val\right)}\right)|X_{\left(val\right)}\right]\\ & \;+Var\left[E\left(y_{\left(val\right)}|y_{p\left(val\right)}\right)|X_{\left(val\right)}\right]\\ & =\sigma_{r\left(te\right)}^{2}+\gamma_{1\left(te\right)}^{2}\sigma_{p\left(val\right)}^{2}, \end{array}$$ [14]

where in the second step of Eq. **14** we again have made the approximation of using the relationship between $y_{\left(val\right)}$ and $y_{p\left(val\right)}$ to model the conditional variance $Var\left[y_{\left(val\right)}|y_{p\left(val\right)},X_{\left(val\right)}\right]$. We show that under the extreme case where the predicted outcome exactly captures the relationship between the outcome and the covariates, we have exactly $Var\left[y_{\left(val\right)}|y_{p\left(val\right)},X_{\left(val\right)}\right]=Var\left[y_{\left(val\right)}|y_{p\left(val\right)}\right]$ (see *SI Appendix*, section 1A.4 for full analytical derivation). Then we estimate the SE of the estimator ${\hat{\beta }}_{\left(val\right)}$ (see *SI Appendix*, section 1B for full analytical derivation):

$$SE\left[{\hat{\beta }}_{\left(val\right)}|X_{\left(val\right)}\right]\approx \sqrt{{\left(X_{\left(val\right)}^{T}X_{\left(val\right)}\right)}^{-1}\left({\hat{\sigma }}_{r\left(te\right)}^{2}+{\hat{\gamma }}_{1\left(te\right)}^{2}{\hat{\sigma }}_{p\left(val\right)}^{2}\right)}.$$ [15]

Therefore, with the estimated corrected coefficient ${\hat{\beta }}_{\left(val\right)}^{⋆}$ and the estimated SE $SE\left({\hat{\beta }}_{\left(val\right)}|X_{\left(val\right)}\right)$, we now can estimate a test statistic to recover the inference we would have made in Eq. **8** when the observed outcomes had been available (see *SI Appendix*, section 1B for details in the hypothesis test and the defined decision rule). The test statistic is approximated as

$$t\left({\hat{\beta }}_{\left(val\right)}\right)\approx \frac{{\hat{\beta }}_{\left(val\right)}^{⋆}}{\sqrt{{\left(X_{\left(val\right)}^{T}X_{\left(val\right)}\right)}^{-1}\left({\hat{\sigma }}_{r\left(te\right)}^{2}+{\hat{\gamma }}_{1\left(te\right)}^{2}{\hat{\sigma }}_{p\left(val\right)}^{2}\right)}}.$$ [16]

### Simulated Data.

We simulate independent covariate x and error term $e_{u}$ and then observe outcome y using the true state of nature model in Eq. **5**. The true state of nature is not directly observed in practical problems but can be specified in simulated problems. We consider both the case of a continuous outcome in *Continuous case* and that of a binary outcome in *Binary case* that demonstrate uncorrected postprediction inference leads to bias in the estimates, small SEs, and anticonservative test statistics.

We also include simulations that demonstrate anticonservative bias in *P* values from uncorrected postprediction inference in *SI Appendix*, section 2A. The key insight of our postpi methods relies on the fitness of the relationship between the observed and predicted outcomes (y and $y_{p}$) estimated in the testing set. In many cases, this relationship can be well described as a simple model but this may not always hold. For instance, when the predicted values are obtained from weak learners, the correlation between the observed and predicted outcomes may not be sufficiently strong to allow corrected inference. As expected, we observe improved operating characteristics of our methods with increasing accuracy of the prediction model. We show that our postpi methods successfully approximate the estimates, SEs, *t* statistics, and *P* values as we would have obtained using the observed y (*SI Appendix*, Figs. 1 and 2). We also show our corrections are reasonably robust to the levels of correlation between y and $y_{p}$ ranging from 0.1 to 0.8. Across all levels of correlation, our postpi methods successfully correct the distribution of *P* values compared to the uncorrected postprediction inference—recovering type I error rate control (*SI Appendix*, Fig. 1).

#### Continuous case.

For the continuous case, we simulate covariates $x_{ij}$ and error terms $e_{u_{i}}$ from normal distributions and then simulate the observed outcome $y_{i}$ using a linear function h(⋅) as the true state of nature model for i=1,…,n, j=1,…,p. (34)

In each simulation cycle, we set the total sample size n=900 and the dimension of covariate matrix p=4. To mimic a complicated data-generating distribution and make predictions sufficiently variable for illustration purposes, we generate data including both linear and smoothed terms. For the smoothed terms, we use Tukey’s running median smoothing with a default smoothing parameter “3RS3R” (35). The error terms are also simulated from a normal distribution with independent variance. The model specification is

$$\begin{array}{cc} x_{i1},x_{i2},x_{i3} & ∼N\left(1,1\right)\\ x_{i4} & ∼N\left(2,1\right)\\ e_{u_{i}} & ∼N\left(0,1\right)\\ y_{i} & =\beta_{1}x_{i1}+\beta_{2}x_{i2}+\beta_{3}\cdot smooth\left(x_{i3}\right)\\ & +\beta_{4}\cdot smooth\left(x_{i4}\right)+e_{u_{i}}. \end{array}$$ [17]

We create a training, testing, and validation set by randomly sampling the observed data into three equal size groups, each with sample size 300. Across the 300 simulated cases, we fix the values of $\beta_{2}=0.5,\beta_{3}=3,\beta_{4}=4$ and set $\beta_{1}$ to be a range of values in [−6,−5,…,5,6] for the covariate of interest $x_{i1}$ in the downstream inferential model. To mimic a more realistic setting, we assume that we are interested only in associating the outcome ($y_{i}$) and one covariate (in this case $x_{i1}$), and we use a linear inference model to quantify this relationship.

For our simulation, we fit a generalized additive model (GAM) (36) to the data in the training set. To estimate the prediction function $\hat{f}\left(\cdot \right)$, we use all of the covariates $x_{i1},x_{i2},x_{i3},x_{i4}$ as features to predict the observed outcomes $y_{i}$. This prediction is meant to simulate the case where we are trying to maximize predictive accuracy, not to perform statistical inference. In the testing set, we apply the trained prediction model to get predicted outcomes $y_{pi}$. We estimate the relationship between the observed and predicted outcome ($y_{i}$ and $y_{pi}$) as a simple linear regression model: $y_{i}∼N\left(\gamma_{0}+\gamma_{1}y_{pi},\sigma_{r}^{2}\right)$.

Our evaluation of the performance of different methods is done on an independent validation set by fitting a linear regression model as the inference model. We compare inference using the predicted outcome with no correction, postprediction inference through analytical derivation postpi and postprediction inference through parametric bootstrap postpi, and nonparametric bootstrap postpi (method details in *SI Appendix*, section 2B). In this simulation, we also have the observed outcome y, so we can calculate the coefficients, estimates, and test statistics that come from using the observed values in inferential models. The baseline model we are comparing to fits the regression model $E\left[y_{i}|x_{i1}\right]=\beta_{0}+x_{i1}\beta_{1}$ to the observed data in the validation set.

**Fig. 4.**
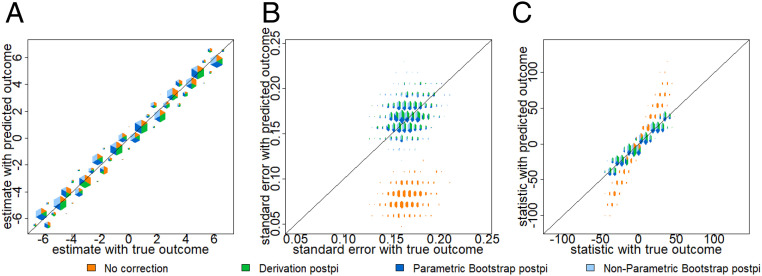
Continuous simulation. Data were simulated from the ground truth model as described in *Continuous case*. On the *x* axis are the values calculated using the observed outcome and on the *y* axis are the values calculated using no correction (orange color), analytical derivation postpi (green color), parametric bootstrap postpi (dark blue color), and nonparametric bootstrap postpi (light blue color). We show (*A*) the estimates are similar across all four approaches since the data were simulated from a normal model, (*B*) the SEs are too small for the uncorrected inference (orange color) but corrected with our approaches, and (*C*) the *t* statistics are anticonservatively biased for uncorrected inference but corrected with our approaches.

We use hextri plots to compare multiple scatter plots simultaneously (37). These plots are designed so that the size of each bin is proportional to the number of points in the bin, and they are divided into colors in proportion to the number of points from each comparison. In this simulation example, the prediction has relatively little bias, so the estimated coefficients using the predicted outcome are relatively close to the estimates using the observed outcome. In Fig. 4*A* all of the colors lie close to the line of equality. However, the SEs for the no correction approach (orange color) in Fig. 4*B* are much lower than what we would have observed in the observed outcomes. This is because the prediction function attempts to capture the mean function, but not the variance in the observed outcome. We compute the root mean-square error (rmse) (38) to show that both the postpi analytical derivation and postpi bootstrap approaches outperform the no correction approach. The SEs are closer to the truth with an rmse reduced from 0.088 for no correction (orange color) to 0.015 for analytical derivation postpi (green color) and also improved to 0.015 for parametric bootstrap postpi (dark blue color) and 0.019 for nonparametric bootstrap postpi (light blue color) in Fig. 4*B*. The improved SEs are reflected in improved *t* statistics using analytical derivation postpi and the two bootstrap postpi approaches in Fig. 4*C*, with rmse reduced from 26.33 for no correction (orange color) to 2.45 for analytical derivation postpi (green color), and improved to 2.41 for parametric bootstrap postpi (dark blue color) and 2.89 for nonparametric bootstrap postpi (light blue color).

#### Binary case.

For the binary case, we simulate a categorical covariate $x_{ic}$, continuous covariates $x_{i1},x_{i2}$, and an error term $e_{u_{i}}$ and then the observed outcome $y_{i}$ assuming a generalized linear model f(⋅) for i=1,…,n. In this case, we specify the true state of nature model f(⋅) to be a logistic regression model. To simulate observed outcomes $y_{i}$, we first set up covariates through a linear combination where we smooth a subset of continuous covariates using Tukey running median smoothing (35) and include errors to increase variability in outcomes $y_{i}$. We apply the inverse logit function to the linear predictor to simulate probabilities which we use to simulate Bernoulli outcomes ($y_{i}=0\;\text{or}\;1$) through binomial distributions. We simulate as follows:

$$\begin{array}{cc} x_{i1} & ∼N\left(1,1\right)\\ x_{i2} & ∼N\left(2,1\right)\\ x_{ic} & ∼Multinom\left(1,\left(1/3,1/3,1/3\right)\right)\\ e_{u_{i}} & ∼N\left(0,1\right)\\ z_{i} & =\beta_{B}1\left(x_{ic}=B\right)+\beta_{C}1\left(x_{ic}=C\right)+\beta_{1}\cdot smooth\left(x_{i1}\right)\\ & +\beta_{2}\cdot smooth\left(x_{i2}\right)+e_{u_{i}}\\ pr_{i} & =\frac{1}{1+e^{-z_{i}}}\\ y_{i} & ∼Binom\left(1,pr_{i}\right). \end{array}$$ [18]

We generate 1,500 samples for each iteration and separate the data into training, testing, and validation sets of equal size *n* = 500. We set $1\left(x_{c}=C\right)$ as the covariate of interest in the subsequent logistic regression inferential model. Then we use the two bootstrap methods—parametric and nonparametric bootstrap postpi—to estimate the corrected coefficient estimate, SE, and test statistic (39).

In the training set, we use a k-nearest neighbors (31) model as a machine-learning tool and all independent covariates $x_{ic},x_{i1},x_{i2}$ as features to estimate the prediction function $\hat{f}\left(\cdot \right)$ . Then we apply the trained prediction model in the testing and validation sets to get the predicted outcome $y_{pi}$ as well as the probability $pr_{i}$ of the predicted outcomes (i.e., $pr_{i}=Pr\left(y_{i}=1\right)$). In the testing set, we use a logistic regression to estimate the relationship between the observed outcome and the predicted probability: $g\left(E\left[y_{i}=1|pr_{i}\right]\right)=\gamma_{0}+pr_{i}\gamma_{1}$, where g(⋅) is the natural log of the odds such that $g\left(p\right)=Ln\left(\frac{p}{1-p}\right)$. Here we form the relationship model with the predicted probability. The reason is that the outcome is dichotomous, so we have little flexibility to model the variance in the observed outcome as a function of the predicted outcome. Instead, using predicted probability provides more flexibility to model the relationship. In the case of a binary outcome, the analytical derivation approach no longer applies, so we apply the two bootstrap correction methods only. In the validation set, we follow the Bootstrap Procedure steps 1 to 5. First, we set the bootstrap size B=100 to start the for loop. In step 3, ii, ${\tilde{y}}_{i}^{b}=k\left(pr_{i}^{b}\right)$, we simulate values in two steps: 1) use $pr_{i}^{b}$ and the estimated relationship model to predict the probability of getting the “success” outcome (i.e., $Pr\left({\tilde{y}}_{i}^{b}=1\right)$) and then 2) sample ${\tilde{y}}_{i}^{b}$ from a binomial distribution with the probability parameter as $Pr\left({\tilde{y}}_{i}^{b}=1\right)$ obtained from step 1. In step 3, iii we again fit a logistic regression model as the inference model: g$\left[E\left({\tilde{y}}_{i}^{b}|x_{c}^{b}\right)\right]=\beta_{p0}+1{\left(x_{c}=C\right)}^{b}\beta_{pC}$. Then in steps 4 and 5 we estimate the parametric and nonparametric bootstrap postpi coefficient, SE, and test statistics.

**Fig. 5.**
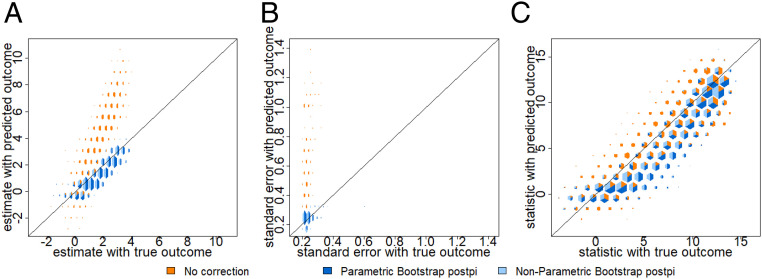
Binary simulation. Data were simulated from the ground truth model as described in *Binary case*. On the *x* axis are the values calculated using the observed outcome and on the *y* axis are the values calculated using no correction (orange color), parametric bootstrap postpi (dark blue color), and nonparametric bootstrap postpi (light blue color). We show (*A*) the uncorrected estimates are anticonservatively biased but this bias is corrected with our postpi approaches, (*B*) the uncorrected SEs are also inflated and corrected by postpi, and (*C*) the *t* statistics show a slight conservative bias compared to the no correction case.

Across the 300 simulated cases, we fix the values of $\beta_{1}=1,\beta_{2}=-2,\beta_{B}=1$. Here we choose $1\left(x_{c}=C\right)$ as the covariate of interest in the downstream inferential analyses, and we set $\beta_{C}$ to be a range of values in [−2,−1.5,…,4.5,5]. Under many simulations, there is a problem of sparsity in the dichotomous covariates where inference from observed $y_{i}$ would be unstable. In this example, we exclude such sparse cases in the simulations which lead to extremely large SEs and inaccurate estimates across all approaches. In Fig. 5 *A* and *B*, we see that the estimates and SEs are inflated in the case of no correction (orange color). In detail, we see bias in the coefficient estimate using the no correction approach (orange color) in Fig. 5*A* with rmse 2.94 compared to the truth. This bias is corrected through the parametric bootstrap (dark blue color) and nonparametric bootstrap (light blue color) postpi methods with rmse reduced to 0.53. The SEs for no correction (orange color) in Fig. 5*B* have rmse 0.49 but reduced to 0.018 for parametric bootstrap postpi (dark blue color) and 0.025 for nonparametric bootstrap postpi (light blue color). In Fig. 5*C*, the *t* statistics have rmse 2.06 using no correction (orange color), 2.04 for parametric bootstrap postpi (dark blue color), and 2.12 for nonparametric bootstrap postpi (light blue color). We observe a slight conservative bias in the *t* statistics due to the postpi corrections—the blue points are consistently slightly below the line of equality. This conservative bias is an acceptable trade-off in cases where the observed outcomes are not available.

### Applications.

To demonstrate the wide applicability of our methodology for performing postprediction inference, we present two examples from very different fields: genomics and verbal autopsy analysis. These applications share very little in common scientifically, but represent two high-profile examples where inference is typically performed with uncorrected predictions as the outcome (dependent) variable.

First, consider the “Recount2” Project (https://jhubiostatistics.shinyapps.io/recount) (40) which consists of RNA sequencing (RNA-seq) gene expression data for over 70,000 human samples aligned using a common pipeline processed in Rail-RNA (41). While Recount2 human samples have available gene expression information, not all samples contain observed phenotype information since the majority of the samples are pulled directly from public data on the sequence read archive (42). However, we previously showed that many of these missing phenotype data can be predicted from the genomic measurements (8). Our goal is to perform inference using these predicted phenotypes.

Second, we describe the distribution of (predicted) causes of death. In regions of the world where routine monitoring of births and deaths is not possible, one approach to estimating the distribution of deaths by cause is the verbal autopsy (VA) survey. These surveys take place with a caregiver or relative of the decedent and ask about the circumstances surrounding the person’s death and typically take place when deaths happen outside of hospitals or routine medical care. Either expert guidance about the relationship between reported symptoms prior to death and the eventual cause or small “gold standard” datasets are used to train algorithms that predict causes of death based on reported symptoms. Algorithm development to predict causes of death is an active area of research and is challenging since data typically contain a mixture of binary, continuous, and categorical symptoms and many causes of death have similar presentations. After assigning a predicted cause of death, a common task is to describe patterns in the cause of death distribution. A scientist may be interested, for example, in how the distribution of deaths varies by region or by sex.

#### Predicting tissue types.

We consider a motivating problem from the Recount2 Project (40) (https://jhubiostatistics.shinyapps.io/recount/). In this example, the phenotype we care about is the tissue type where the RNA is sampled from (43). Understanding gene expression levels across tissues and cell types has many applications in basic molecular biology. Many research topics concentrate on finding which genes are expressed in which tissues, aiming to expand our fundamental understanding of the origins of complex traits and diseases (44–48). The Genotype-Tissue Expression (GTEx) project (49), for example, studies how gene expression levels are varied across individuals and diverse tissues of the human body for a wide variety of primary tissues and cell types (44, 49). Therefore, to better understand the cellular process in human biology, it is important to study the variations in gene expression levels across tissue types.

Even though tissue types are available in GTEx (49), they are not available for most samples in the Recount2. In a previous paper (8), we developed a method to predict for those missing phenotypes using gene expression data. In this example, we collected a subset of samples that we have observed tissue types as breast or adipose tissues. We also had predicted values for the above samples calculated in a previous training set (8) using the 2,281 expressed regions (50) as predictors. Our goal in this example is to understand which of these regions are most associated with breast tissue in new samples (i.e., samples without observed tissue types) so that we can understand which measured genes are most impacted by the biological differences between breast and adipose tissues. Although here the phenotype we care about is the tissue types, especially breast and adipose tissues, our method can be broadly applied to any predictions to all phenotypes.

To test our method, we collected 288 samples from the Recount2 with both observed and predicted tissue types. Among the observed tissue types, 204 samples are observed as adipose tissues and 84 samples are observed as breast tissues. The predicted values obtained from a previously trained dataset (8) include the predicted tissue type (i.e., adipose tissue or breast tissue) and the probability for assigning a predicted tissue type. In this example, we compare no correction and postpi bootstrap approaches only since the outcomes (tissue types) we care about are categorical.

The inference model we are interested in is $g\left[E\left(y_{i}=1|ER_{i}^{j}\right)\right]=\beta_{0}^{j}+\beta_{1}^{j}ER_{j}$. Here g(⋅) is the logit link function for j=1,…,2,281 (expressed regions) and i=1,…,n, n is the total number of samples in the Recount2. In the model, $y_{i}=1$ or $y_{i}=0$ represents whether breast tissue is observed or adipose tissue is observed at the ith sample, and $ER_{i}^{j}$ is the gene expression level for the jth region on the ith sample.

For this dataset (288 samples), we have binary tissue type outcomes. Since the predicted outcomes were obtained in a previously trained set (8), we need only to separate our data into a testing and a validation set, each with a sample size n=144. In the testing set, we fit a k-nearest neighbors (31) model to estimate the relationship between the observed tissue type and the probability of assigning the predicted value. In the validation set, we follow the Bootstrap Procedure. Particularly in step 3, ii, we simulate values from a distribution ${\tilde{y}}_{i}^{b}|pr_{i}^{b}∼F_{\hat{\gamma }}$. Similar to what we did with the simulated data in *Simulated Data*, in this example, we set $F_{\gamma }$ to be a binomial distribution with the probability parameter (i.e., probability of assigning the outcome as breast cancer) estimated from the relationship model. In this way, we utilize the estimated relationship to account for necessary variations in simulated outcomes.

Among the 2,281 expressed regions (50) used to make tissue type predictions (8), we care about the regions that have expression values across a relatively large amount of samples in the validation set. It is a well-known phenomenon that many RNA-seq measurements may be zero if the number of collected reads is low. To avoid highly variable model fits due to zero variance covariates, we only fit logistic regressions inference models to each filtered expressed region with expressed values over at least 20% of samples. Under this filtering procedure, we include 101 expressed regions as regression variables and fit the inference model described above to each region in the validation set. We then get 101 estimates, SEs, and *t* statistics. We compare them to the no correction approach as we did with the simulated data.

**Fig. 6.**
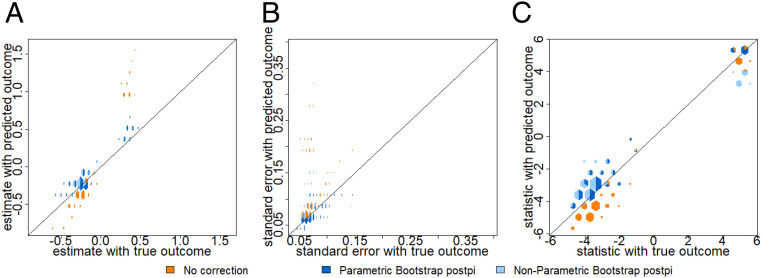
Breast versus adipose tissue prediction. Data were collected from the Recount2 as described in *Predicting tissue types*. On the *x* axis are the values calculated using the observed outcome and on the *y* axis are the values calculated using no correction (orange color), parametric bootstrap postpi (dark blue color), and nonparametric bootstrap postpi (light blue color). We show (*A*) the estimates, (*B*) the SEs, and (*C*) the *t* statistics. The two bootstrap postpi approaches clearly improve the estimates and SEs compared to no correction.

By comparing the rmse, we observed that the estimates, SEs, and test statistics are improved from no correction to parametric and nonparametric bootstrap postpi methods. In Fig. 6*A*, no correction (orange color) estimates have rmse 0.36 compared to the truth and it reduces to 0.08 with parametric bootstrap postpi (dark blue color) and nonparametric bootstrap postpi (light blue color). The SEs in Fig. 6*B* have rmse 0.08 for no correction (orange color), but corrected to 0.01 for parametric bootstrap postpi (dark blue color) and 0.03 for nonparametric bootstrap postpi (light blue color). The resulting *t* statistics are improved from rmse 0.91 for no correction (orange color) to 0.63 for parametric bootstrap postpi (dark blue color) and 0.93 for nonparametric bootstrap postpi (light blue color).

We also applied our approach to correct inference for models using predicted RNA quality as an example of how to do postprediction inference for continuous outcomes (*SI Appendix*, section 3A).

#### Describing cause of death distributions.

We now move to our second example where the outcome of interest is the (predicted) cause of death and inputs are symptoms or circumstances reported by a caregiver or relative (51). Symptoms might include, for example, whether a person had a fever before the person died, how long a cough lasted (if one was reported), or the number of times the person visited a medical professional. We use data from the Population Health Metrics Research Consortium (PHMRC), which consists of about 7,800 gold standard deaths from six regions around the world. These data are rare because they contain both a physical autopsy (including pathology and diagnostic testing) and a verbal autopsy survey. Typically, only a small fraction of deaths will have an assigned cause (e.g., by a clinician reading the verbal autopsy survey) and these few labeled deaths will be used as inputs to train a model for the remaining deaths.

We split the data into training and testing sets, with 75% of the data used for training. The PHMRC data classify cause of death at several levels of granularity. For our experiments, we combined causes into 12 broad causes of death (cancers, diabetes, renal diseases, liver diseases, cardiovascular causes, stroke, pneumonia, HIV/AIDS or tuberculosis, maternal causes, external causes, other communicable diseases, and other noncommunicable diseases). We predicted the cause of death using *InSilicoVA* (52) which uses a naive Bayes classifier embedded in a Bayesian framework to incorporate uncertainty between cause classifications.

In this example, we want to understand trends in the 12 combined causes of death across multiple symptoms representing health behaviors and demographics. Demographic symptoms include age of the decedent and sex (male or female) of the decedent. Behavioral symptoms include whether the decedent used tobacco (yes or no), used alcohol (yes or no), and used medical care for the illness (yes or no). Additional symptoms include whether the decedent had obesity (yes or no), accident (yes or no), and previous health records (yes or no). These symptoms are used in the training model as a subset of the symptoms to classify the cause of death with *InSilicoVA* (52) and used again for downstream statistical inference. The inference model we are interested in is $g\left[E\left(y_{i}|SYM_{i}^{\;j}\right)\right]=\beta_{0}^{j}+\beta_{1}^{j}SYM_{j}$. Here g(⋅) is the logit link function for j=1,…,13 (symptoms) and i=1,…,n, n is the total number of samples in the dataset. In this model, $y_{i}$ represents one of the 12 combined causes at the ith sample and $SYM_{i}^{\;j}$ is the jth symptom of interest on the ith sample.

For this dataset, we use categorical outcomes as the causes of death for the 1,960 samples and assume the outcomes are unobserved, as they typically would be in practice, for the remaining cases. Since the predicted values were obtained in a previously trained set using *InSilicoVA* (52), we separate our data only into testing and validation sets, each with a sample size n=980. In the testing set, we fit a k-nearest neighbors model (31) to estimate the relationship between the observed cause of death and the probability of assigning the cause. In the validation set, we follow the Bootstrap Procedure. Particularly in step 3, ii, we simulate values from a distribution ${\tilde{y}}_{i}^{b}|pr_{i}^{b}∼F_{\hat{\gamma }}$. In this example, we set $F_{\gamma }$ to be a multinomial distribution with the probability parameters (i.e., probability of assigning each of the 12 broad causes of death) estimated from the relationship model as we did in the simulated data.

Among all of the symptoms used to make causes of death prediction (52), we use a subset of symptoms that also have balanced classes across the 12 broad causes of death. This is to avoid highly variable model fits due to zero variance covariates that is categorized as a well-known issue for sparse outcomes (53). We then filter the eight symptoms we are interested in as regression variables and fit a logistic regression inference model to each selected symptom in the validation set. There is one continuous variable and there are seven categorical regression variables, each with two factor levels (yes or no). For the inference results, we get eight estimates, SEs, and *t* statistics in the validation set. We then compare them to the no correction approach as we did with the simulated data.

**Fig. 7.**
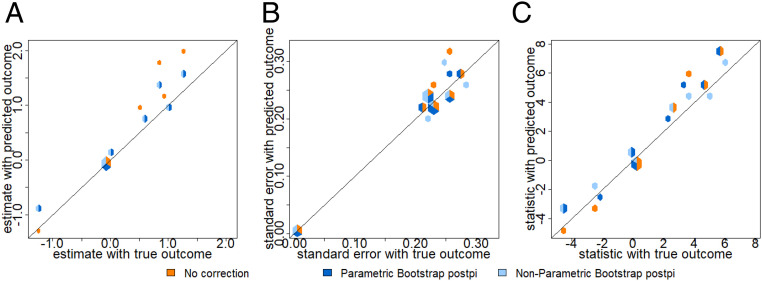
Twelve causes of death prediction. Data were collected from PHMRC described in *Describing cause of death distributions*. On the *x* axis are the values calculated using the observed outcome and on the *y* axis are the values calculated using no correction (orange color), parametric bootstrap postpi (dark blue color), and nonparametric bootstrap postpi (light blue color). We show (*A*) the estimates, (*B*) the SEs, and (*C*) the *t* statistics. The parametric bootstrap postpi approach improves the rmse of estimates, SEs, and *t* statistics compared to no correction.

We observed that the uncorrected estimates, SEs, and *t* statistics (orange color) have higher rmse compared to the parametric bootstrap postpi method (dark blue color). In Fig. 7*A* the no correction estimates have rmse 0.46 (orange color) compared to the truth, which is reduced to 0.24 with parametric (dark blue color) and nonparametric (light blue color) bootstrap postpi methods. The no correction SEs in Fig. 7*B* have a rmse of 0.03 (orange color), which are corrected to rmses of 0.013 for parametric bootstrap postpi (dark blue color) and 0.024 for the nonparametric bootstrap postpi (light blue color). The resulting *t* statistics in Fig. 7*C* are improved from an rmse of 1.21 for no correction (orange color) to 0.79 for parametric bootstrap postpi (dark blue color) and to 0.73 for nonparametric bootstrap postpi (light blue color).

## Discussion

As machine learning becomes more common across a range of scientific settings, predicted outcomes will be used more often as dependent variables in subsequent statistical analyses. As we have shown, an uncorrected postprediction inference can lead to highly variable or biased estimates of parameters of interest, SEs that are too small, anticonservatively biased *P* values, and false positives.

We introduced methods to correct for postprediction inference and adjust point and interval estimates when using predicted outcomes in place of observed outcomes. Our method is flexible enough to be applied to continuous and categorical outcome data, observed in fields such as medicine, public health, and sociology. Through simulated and real data, we show that our results outperform the most common current approach of ignoring the prediction step and performing inference without correction. By appropriately modeling the variability and bias due to the prediction step, the estimates, SEs, test statistics, and *P* values are corrected toward the gold standard analysis we would obtain as if we had used the true outcomes.

Our approach relies on the key observation that the relationship between the observed and predicted outcomes can be described as a simple model. While this observation is empirically true for the models and algorithms we considered, it may not hold universally. One limitation of our approach is that it depends on the fitness of the relationship model. For instance, when the predicted values are obtained from weak learners, the correlation between the observed and predicted outcomes is not strong, which may not be well captured by a simple model. Another limitation is that we assume the training, testing, and validation sets follow the same data-generating distribution. If this assumption does not hold, inference performed on the bootstrapped values in the validation set will no longer reflect the true underlying data-generating process. A potential solution is that we should first conduct data normalization using methods such as surrogate variable analysis (54), remove unwanted variation (55), and removeBatchEffect in linear models for microarray data (56) to correct for latent confounders in the testing or validation sets. The normalized samples can then be input into our method for subsequent inferential analyses.

Despite these limitations, correcting for postprediction inference is crucial for accurate inference when using outcomes produced by machine-learning methods. Our correction represents a step toward a general solution to the postprediction inference problem.

## Supplementary Material

Supplementary File

## Data Availability

RNA-seq, verbal autopsy, and simulation data have been deposited in postpi (https://osf.io/g4w28/). To make this method useable by the community we have released the postpi R package: https://github.com/leekgroup/postpi.
