# Supplementary material for: Methods for correcting inference based on outcomes predicted by machine learning
Source: Proc Natl Acad Sci U S A. 2020 Nov 18;117(48):30266–75. doi: 10.1073/pnas.2001238117 (PMC7720220; doi:10.1073/pnas.2001238117)
Supplement: Supplementary File [file pnas.2001238117.sapp.pdf]

Supplement information for methods for correcting inference based on outcomes predicted by machine learning

Siruo Wang[a], **Tyler H. McCormick**[b,c], **and Jeffrey T. Leek**[a,1]

[a]Department of Biostatistics, Johns Hopkins Bloomberg School of Public Health, Baltimore, MD 21205, USA; [b]Departments of Statistics, University of Washington, Seattle, WA 98195, USA; [c]Departments of Sociology, University of Washington, Seattle, WA 98195, USA

DRAFT

1. Analytical derivation

In the following analytical derivations, we denote observed outcomes as y, predicted outcomes as y_p, covariates as x, and covariates in matrix notation as X. We assume that the data have been divided into training (tr), testing (te), and validation sets (val) and that the data generating distribution is the same across the three sets: $y \sim N(f(x), \sigma_t^2)$ where $f(\cdot)$ is an arbitrary and unknown function of the covariates. Throughout the supplement, we use abbreviations for the testing, training, and validation sets when values are specific to those sets but omit them when the statements apply to all three. We also keep subscripts on β to differentiate between sets but throughout assume $\beta = \beta_{(val)} = \beta_{(te)} = \beta_{(tr)}$. The estimated values of these coefficients, however, do differ across the three sets. We are interested in inference for the simplified model: $y \sim N(X\beta, \sigma^2)$. This represents common statistical practice where the complicated underlying functional relationship between the outcome and covariates is unknown but a regression modeling strategy is employed to perform inference. Under our problem setup the observed data consist of:

- The observed outcomes and covariates in the training set $(y_{(tr)}, x_{(tr)})$
- A prediction function estimated based on the training data $y_{p(tr)} = \hat{f}(x)$
- The observed outcomes, predicted outcomes, and observed covariates in the test set $(y_{(te)}, y_{p(te)}, x_{(te)})$
- The predicted outcomes and observed covariates in matrix notation in the validation set and beyond $(y_{p(val)}, X_{(val)})$

Our goal is to infer the relationship between the outcome y and some subset of the covariates in the validation set or a future data set where the collection of outcome is either prohibitively expensive or complicated.

In this validation set ideally we would fit the model:

$$y_{(val)}|X_{(val)} \sim N(X_{(val)}\beta_{(val)}, \sigma_i^2). \quad [1]$$

However, the outcome is not observed in the validation set. We therefore instead will fit the model:

$$y_{p(val)}|X_{(val)} \sim N(X_{(val)}\beta_{p(val)}, \sigma_p^2) \quad [2]$$

where $\beta_{p(val)}$ corresponds to the coefficient estimated when using the predicted outcome y_p rather than the observed outcome. Our goal is to recover the inference we would have made as if the observed outcomes had been available.

As we point out in the main text in Section 3.3, we can use information about the relationship between the observed outcome $y_{(te)}$ and predicted outcome $y_{(te)p}$ in the testing set to correct inference in data sets where $y_{(val)}$ is not observed and we substitute $y_{p(val)}$. We make the assumption that the observed outcomes and predicted outcomes follow a relationship model:

$$y_{(te)} \sim N(\gamma_0 + \gamma_1 y_{p(te)}, \sigma_r^2). \quad [3]$$

We can estimate the coefficients from the testing set where both $y_{(te)}$ and $y_{p(te)}$ are available and then assume that the relationship also holds in the validation set where we only have access to $y_{(val)p}$. The key insight we have made is that a simplified model often holds, even when the machine learning function used to make the predictions $\hat{f}(x)$ is quite complicated (see main text Figure 3).

A. Conditional expectation and variance.

A.1. Approximating the conditional distribution of y. In the validation set, we have assumed that only the covariates $(x_{(val)})$ and predicted outcomes $(y_{p(val)})$ are available. Critically $y_{(val)}$ is not available. As a first step, we propose an approximation of the conditional expectation of the unobserved outcome $y_{(val)}$ given covariates of interest in matrix notation $X_{(val)}$ and the predicted outcomes $y_{p(val)}$ by leveraging the assumed relationship model $y \sim N(\gamma_0 + \gamma_1 y_p, \sigma_r^2)$ and the linear inferential model based on using the predicted outcomes $y_p \sim N(X\beta_p, \sigma_p^2)$. We approximate the conditional distribution of the unobserved $y_{(val)}$ as follows:

$$\begin{aligned} E(y_{(val)}|X_{(val)}) &= E\left[E(y_{(val)}|X_{(val)}, y_{p(val)})|X_{(val)}\right] \\ &\approx E\left[E(y_{(val)}|y_{p(val)})|X_{(val)}\right] \\ &= E\left[\gamma_{0(val)} + \gamma_{1(val)} y_{p(val)}|X_{(val)}\right] \\ &= \gamma_{0(val)} + \gamma_{1(val)} E(y_{p(val)}|X_{(val)}) \\ &= \gamma_{0(val)} + \gamma_{1(val)}\ X_{(val)}\beta_{p(val)} \end{aligned} \quad [4]$$

where the approximation in equation (4) is based on using the relationship between the predicted outcome and observed outcome $E(y_{(val)}|y_{p(val)})$ as an approximation to the conditional expectation $E(y_{(val)}|X_{(val)}, y_{p(val)})$. Since the predicted values $y_{p(val)}$ and the design matrix $X_{(val)}$ are assumed to be observed in the validation set, we can estimate $\beta_{p(val)}$ using the validation data. However, the estimation of γ_0, γ_1 requires

[1]To whom correspondence should be addressed. E-mail: jtleek@gmail.com

both the observed and predicted outcomes. By assumption, we observe both of these values in the testing set, and we can therefore write:

$$E(y_{(val)}|X_{(val)}) \approx \gamma_{0(val)} + \gamma_{1(val)}\ X_{(val)}\beta_{p(val)} = \gamma_{0(te)} + \gamma_{1(te)}\ X_{(val)}\beta_{p(val)} \quad [5]$$

Where the equality in equation (5) follows from the assumption that the data generating distribution in the testing and validation sets is the same.

A.2. Approximating the coefficients. We want to fit a linear regression model as the inferential model between $y_{(val)}$ and $X_{(val)}$. This model can be written as $y_{(val)} \sim N(X_{(val)}\beta_{(val)}, \sigma_i^2)$. In the validation set, we assume that the true outcomes $y_{(val)}$ are unobserved, our goal is to correctly estimate $\beta_{(val)}$ using the relationship model in the testing set, predicted outcome and covariates in the validation set.

If we had observed $y_{(val)}$, we have the OLS estimator $\hat{\beta}_{(val)} = (X^T_{(val)}X_{(val)})^{-1}X^T_{(val)}y_{(val)}$. Using the conditional expectation for $y_{(val)}$ that we have previously computed and the relationship model from the testing set, we have $E(y_{(val)}|X_{(val)}) \approx \gamma_{0(te)} + \gamma_{1(te)}X_{(val)}\beta_{p(val)}$, and thus we can estimate the unobserved outcome $y_{(val)}$ as:

$$y^\star_{(val)} = \hat{\gamma}_{0(te)} + \hat{\gamma}_{1(te)}X_{(val)}\hat{\beta}_{p(val)} \quad [6]$$

Therefore, we can approximate the estimator $\hat{\beta}_{(val)}$ as:

$$\hat{\beta}^\star_{(val)} = (X^T_{(val)}X_{(val)})^{-1}X^T_{(val)}(\hat{\gamma}_{0(te)} + \hat{\gamma}_{1(te)}X_{(val)}\hat{\beta}_{p(val)}) \quad [7]$$

The bias of this estimator can be computed as:

$$\begin{aligned} &E(\hat{\beta}^\star_{(val)} - \beta_{(val)}|X_{(val)}) \\ &= E[(X^T_{(val)}X_{(val)})^{-1}X^T_{(val)}(\hat{\gamma}_{0(te)} \\ &\quad + \hat{\gamma}_{1(te)}X_{(val)}\hat{\beta}_{p(val)})|X_{(val)}] - \beta_{(val)} \\ &= (X^T_{(val)}X_{(val)})^{-1}X^T_{(val)}E(y^*_{(val)}|X_{(val)}) - \beta_{(val)} \end{aligned} \quad [8]$$

where $y^*_{(val)}$ is our approximation to y. This expectation is a complicated function of the training, testing, and validation sets, but for good prediction functions will have expectation approximately equal to $y_{(val)}$ - which would lead to nearly unbiased estimation of $\beta_{(val)}$. We observe this behavior in the simulated examples in Section 2 of this supplement.

A.3. Conditional variance. The analytical derivation of the conditional variance of unobserved outcome $y_{(val)}$ given covariate of interest $X_{(val)}$ in the validation set can be estimated using the variance that comes from both the relationship model $y \sim N(\gamma_0 + \gamma_1 y_p, \sigma_r^2)$ and the linear inferential model $y_p \sim N(X\beta_p, \sigma_p^2)$ using a similar approach to the analytical derivation above where we assume that the observed outcome is unknown. Using the law of total conditional variance:

$$\begin{aligned} &Var(y_{(val)} \mid X_{(val)}) \\ &= E\left[Var(y_{(val)} \mid y_{p(val)}, X_{(val)}) \mid X_{(val)}\right] \\ &\quad + Var\left[E(y_{(val)} \mid y_{p(val)}, X_{(val)}) \mid X_{(val)}\right] \\ &\approx E\left[Var(y_{(val)} \mid y_{p(val)}) \mid X_{(val)}\right] \\ &\quad + Var\left[E(y_{(val)} \mid y_{p(val)}) \mid X_{(val)}\right] \\ &= E(\sigma^2_{r(val)} \mid X_{(val)}) \\ &\quad + Var(\gamma_{0(val)} + \gamma_{1(val)}y_{p(val)} \mid X_{(val)}) \\ &= \sigma^2_{r(val)} + \gamma^2_{1(val)}Var(y_{p(val)} \mid X_{(val)}) \\ &= \sigma^2_{r(val)} + \gamma^2_{1(val)}\sigma^2_{p(val)} \end{aligned} \quad [9]$$

Where the approximation in Equation (9) is the same as the conditional expectation estimated above.

Since the observed $y_{(val)}$ is not available in the testing set, we can approximate the variance by borrowing estimates from the testing set for the γ coefficients:

$$Var(y_{(val)} \mid X_{(val)}) \approx \sigma^2_{r(val)} + \gamma^2_{1(val)}\sigma^2_{p(val)} = \sigma^2_{r(te)} + \gamma^2_{1(te)}\sigma^2_{p(val)} \quad [10]$$

where the equality in Equation (10) follows from the assumption that the data generating distribution in the testing and validation sets is the same.

A.4. Extreme case. Under the extreme case where the predicted outcome exactly captures the relationship between the outcome and the covariates $y_p = f(x)$, then the real outcome can be written as $y = y_p + \epsilon = f(x) + \epsilon$. In this setting, we can show that the approximation based on the relationship model in the conditional expectation analytical derivation in section A.1 and the conditional variance analytical derivation in section A.3 can be replaced with equality. In detail, we want to show that under the extreme case, $E[E(y|y_p, X)|X] = E[E(y|y_p)|X]$, $E[Var(y|y_p, X)|X] = E[Var(y|y_p)|X]$, and $Var[E(y|y_p, X)|X] = Var[E(y|y_p)|X]$.

Consider the simplest case where $f(X) = X\beta$, $y = X\beta + \epsilon$, and $y_p = X\beta$. We also assume $\epsilon \sim N(0, \sigma^2)$, $X \sim N(\delta, \Sigma)$. Then, we write the assumptions as follows:

$$\begin{aligned} &y|X \sim N(X\beta, \sigma^2) \\ &y_p|X \sim N(X\beta, 0) \\ &X \sim N(\delta, \Sigma) \end{aligned} \quad [11]$$

The marginal distribution of y and y_p is written as:

$$\begin{aligned} &y \sim N(\delta\beta, \beta^T\Sigma\beta + \sigma^2) \\ &y_p \sim N(\delta\beta, \beta^T\Sigma\beta) \end{aligned} \quad [12]$$

Therefore, $\begin{pmatrix} y \\ y_p \end{pmatrix}$ can be written as a bivariate normal distribution:

$$N\left(\begin{pmatrix} \delta\beta \\ \delta\beta \end{pmatrix}, \begin{pmatrix} \beta^T\Sigma\beta + \sigma^2 & \rho(\beta^T\Sigma\beta + \sigma^2)^{1/2}(\beta^T\Sigma\beta)^{1/2} \\ \rho(\beta^T\Sigma\beta + \sigma^2)^{1/2}(\beta^T\Sigma\beta)^{1/2} & \beta^T\Sigma\beta \end{pmatrix} \quad [13]$$

DRAFT

Here ρ is the correlation between y and y_p, and $\rho = \frac{(\beta^T\Sigma\beta)^{1/2}}{(\beta^T\Sigma\beta+\sigma^2)^{1/2}}$. To see this note that:

$$\begin{aligned} Cov(y, y_p) &= E(yy_p) - E(y)E(y_p) \\ &= E(y_p^2 + \epsilon y_P) - (\delta\beta)^2 \\ &= E(y_p^2) - (\delta\beta)^2 \\ &= Var(y_p) + E(y_p)^2 - (\delta\beta)^2 \\ &= \beta^T\Sigma\beta \end{aligned} \quad [14]$$

and thus

$$\begin{aligned} \rho &= \frac{\beta^T\Sigma\beta}{(\beta^T\Sigma\beta)^{1/2}(\beta^T\Sigma\beta+\sigma^2)^{1/2}} \\ &= \frac{(\beta^T\Sigma\beta)^{1/2}}{(\beta^T\Sigma\beta+\sigma^2)^{1/2}} \end{aligned} \quad [15]$$

We want to show that $E[E(y|y_p, X)|X] = E[E(y|y_p)|X]$. First, based on what we assume that both y and y_p capture all signals of X, we see:

$$E(y|y_p, X) = E(y|X) = X\beta \quad [16]$$

so, the left side of equation is:

$$E[E(y|y_p, X)|X] = E(X\beta|X) = X\beta \quad [17]$$

Second, based on the bivariate normal distribution of y and y_p provide above, we have:

$$\begin{aligned} E(y|y_p) &= \delta^T\beta + \rho\frac{(\beta^T\Sigma\beta+\sigma^2)^{1/2}}{(\beta^T\Sigma\beta)^{1/2}}(y_p - \delta^T\beta) \\ &= \delta^T\beta + \frac{(\beta^T\Sigma\beta)^{1/2}}{(\beta^T\Sigma\beta+\sigma^2)^{1/2}}\frac{(\beta^T\Sigma\beta+\sigma^2)^{1/2}}{(\beta^T\Sigma\beta)^{1/2}}(y_p - \delta^T\beta) \\ &= \delta^T\beta + (y_p - \delta^T\beta) \\ &= y_p \end{aligned} \quad [18]$$

so, the right side of equation is:

$$E[E(y|y_p)|X] = E(y_p|X) = X\beta \quad [19]$$

This demonstrates that both expectations are equal so:

$$E[E(y|y_p, X)|X] = E(y|y_p)|X] = X\beta \quad [20]$$

We also want to show that under the extreme case scenario, we have $Var[E(y|y_p, X)|X] = Var[E(y|y_p)|X]$ and $E[Var(y|y_p, X)|X] = E[Var(y|y_p)|X]$. First, we show $Var[E(y|y_p, X)|X] = Var[E(y|y_p)|X]$ holds true using the expectations $E(y|y_p, X)$ and $E(y|y_p)$ calculated above. On the left side of equation, we see:

$$Var[E(y|y_p, X)|X] = Var(X\beta|X) = 0 \quad [21]$$

On the right side of equation, we see:

$$Var[E(y|y_p)|X] = Var(y_p|X) = 0 \quad [22]$$

Thus, we have

$$Var[E(y|y_p, X)|X] = Var[E(y|y_p)|X] = 0 \quad [23]$$

Second, to show $E[Var(y|y_p, X)|X] = E[Var(y|y_p)|X]$, we can show $Var(y|y_p, X) = Var(y|y_p)$. On the left side of equation,

$$Var(y|y_p, X) = Var(y|X) = \sigma^2 \quad [24]$$

On the right side of the equation, we use the variance from the bivariate normal distribution of y and y_p,

$$\begin{aligned} Var(y|y_p) &= (1-\rho^2)(\beta^T\Sigma\beta+\sigma^2) \\ &= \left(1 - \frac{\beta^T\Sigma\beta}{\beta^T\Sigma\beta+\sigma^2}\right)(\beta^T\Sigma\beta+\sigma^2) \\ &= \sigma^2 \end{aligned} \quad [25]$$

Thus, we have

$$Var(y|y_p, X) = Var(y|y_p) = \sigma^2 \quad [26]$$

and

$$E[Var(y|y_p, X)|X] = E[Var(y|y_p)] = E(\sigma^2|X) = \sigma^2 \quad [27]$$

In general, we do not expect the machine learning prediction $\hat{f}(x)$ to perfectly estimate the entire signal in y, however, this extreme case serves to illustrate that the more the machine learning prediction captures the signal in the data, the closer our approximation will be. This is substantiated both by the observation that a variety of different machine learning models appear to make predictions that follow the relationship model relatively closely (see main text Figure 2) and our simulations, which show improved performance of our analytical derivation approximation as the accuracy of the machine learning model increases (see supplement Figure 1 and Figure 2).

B. Test statistic. In Section 1A.1, we estimate the corrected coefficient $\hat{\beta}^{\star}_{(val)} = (X^T_{(val)}X_{(val)})^{-1}X^T_{(val)}(\hat{\gamma}_{0(te)} + \hat{\gamma}_{1(te)}X_{(val)}\hat{\beta}_{p(val)})$ and in Section 1A.3 we estimate the corrected conditional variance $Var(y_{(val)}|X_{(val)}) = \sigma^2_{r(te)} + \gamma^2_{1(te)}\sigma^2_{p(val)}$. The corrected coefficient and conditional variance improve from the no correction values which are calculated directly in the linear inferential model using the predicted outcome $y_{p(val)}$. The corrected values are more close to the gold standard coefficient and conditional variance as if we are using the real outcomes $y_{(val)}$ in the inference model.

To form a test statistic for the corrected coefficient $\hat{\beta}_{(val)}$, we also need the standard error of $\hat{\beta}_{(val)}$. If real outcome $y_{(val)}$ are observed in the validation set and we use $y_{(val)}$ to fit a linear regression model as the inference model, we have:

$$Var(\hat{\beta}_{(val)}|X_{(val)}) = (X^T_{(val)}X_{(val)})^{-1}Var(y_{(val)}|X_{(val)}) \quad [28]$$

Because in real settings the real outcome $y_{(val)}$ are unobserved, we use: $\hat{\sigma}^2_{r(te)} + \hat{\gamma}^2_{1(te)}\hat{\sigma}^2_{p(val)}$ to approximate $Var(y_{(val)}|X_{(val)})$ (see details in section 1A.3), and then $Var(\hat{\beta}_{(val)}|X_{(val)})$ can be approximated as:

$$Var(\hat{\beta}_{(val)}|X_{(val)}) \approx (X^T_{(val)}X_{(val)})^{-1}(\hat{\sigma}^2_{r(te)} + \hat{\gamma}^2_{1(te)}\hat{\sigma}^2_{p(val)}) \quad [29]$$

and then,

$$se(\hat{\beta}_{(val)}|X_{(val)}) \approx \sqrt{(X^T_{(val)}X_{(val)})^{-1}(\hat{\sigma}^2_{r(te)} + \hat{\gamma}^2_{1(te)}\hat{\sigma}^2_{p(val)})} \quad [30]$$

Using the estimated corrected coefficient $\hat{\beta}^{\star}_{(val)}$ and the estimated standard error $se(\hat{\beta}_{(val)}|X_{(val)})$, we now are able to

DRAFT

estimate a test statistic to recover the inference we would have made in the regression model Equation (1), while we actually fit the model Equation (2). To test for the null hypothesis against the alternative of the form: $H_0 : \beta_{(val)k} = 0$ vs. $H_a : \beta_{(val)k} \neq 0$ ($\beta_{(val)k}$ is the k-th component of the estimator vector), we can then estimate the test statistic as:

$$t(\hat{\beta}_{(val)}) \approx \frac{\hat{\beta}^{\star}_{(val)}}{\sqrt{(X^T_{(val)} X_{(val)})^{-1}(\hat{\sigma}^2_{r(te)} + \hat{\gamma}^2_{1(te)}\hat{\sigma}^2_{p(val)})}} \quad [31]$$

We define a decision rule to decide whether the null hypothesis shall be rejected or not. One way is to compare the test statistic. We reject the null hypothesis $H_0 : \beta_{(val)k} = 0$ in favor of the alternative hypothesis $H_a : \beta_{(val)k} \neq 0$ at the significance level α when $t(\hat{\beta}_{(val)k}) > t^{\alpha}_{n-p}$, where t^{α}_{n-p} is from the t statistical table with p degrees of freedom and significance level α.

2. Simulation

A. Simulation with increasing correlation between predicted and observed outcomes. In this section, we show a comparison of no correction, analytical derivation postpi, parametric bootstrap postpi, and non-parametric bootstrap postpi methods in the simulated data where the correlations between the predicted and observed outcomes are different.

First, we simulate continuous covariates x_{ij} and error terms e_{u_i} from normal distributions and simulate the observed outcome y_i using a nonlinear function - a combination of smoothed (1) terms, quadratic and cubic terms. The model specification is:

$$\begin{aligned} x_{i1}, x_{i2}, x_{i3} &\sim \mathcal{N}(1,1) \\ e_{u_i} &\sim \mathcal{N}(0,1) \\ y_i &= \beta_1 x_{i1} + \beta_2 x_{i2}^2 + \beta_3 \cdot smooth\,(x_{i3})^3 \\ &\quad + smooth\left(e^2_{u_i}\right) \end{aligned} \quad [32]$$

In each simulation cycle, we set the total sample size $n = 900$, and then create a training, testing, and validation set by randomly sampling the observed data into three equal-size groups each with sample size 300.

In the training set, we fit a generalized additive model (GAM) (2) to estimate the prediction function $\hat{f}(\cdot)$ and we use all of the covariates x_{i1}, x_{i2}, x_{i3} as features to predict the observed outcomes y_i. In the testing set, we apply the trained prediction model to get predicted outcomes y_{pi}. We estimate the relationship between the observed and predicted outcome (y_i and y_{pi}) as a simple linear regression model: $y_i \sim N(\gamma_0 + \gamma_1 y_{pi}, \sigma^2_r)$. In the validation set, we will use a linear inference model to quantify this relationship between predicted outcome y_{pi} and covariate of interest x_{1i}.

Across 300 simulated cases, we fix the values of $\beta_2 = 1.5, \beta_3 = 1.5$ and set the standard error of the error term e_{u_i} to be a range of values in $[0, 0.5, 1, ..., 6.5, 7]$. By changing this value, we can control the correlation between the observed and predicted outcomes in the testing set, to evaluate the performance of different methods. In the following two simulation cases, we set $\beta_1 = 0$ and $\beta_1 = 2$. In each case, we compute the estimates, standard errors, and t-statistics for β_1 with no correction, analytical derivation postpi, parametric, and non-parametric bootstrap approaches. Then we evaluate the performance of the above three different methods by comparing the results to the baseline results where the true outcome is observed.

In the first simulation example in Figure 1, we set $\beta_1 = 0$ and compare the performance of the no correction and three postpi methods to the truth (baseline results where the outcome is observed in the validation set) under the correlation between predicted and observed outcome to be 0.1 - 0.2 to 0.7 - 0.8. The prediction has relatively little bias, so the estimated coefficients using the predicted outcome are relatively close to the estimates using the observed outcome in Figure 1(a) no matter the correlation between y and y_p is small or large. However, the standard errors for the no correction approach (orange color) in Figure 1(b) is much lower than what we would have observed in the observed outcomes. This is because the prediction function attempts to capture the mean function, but not the variance in the observed outcome. We show that the derivation postpi (green color), parametric bootstrap postpi (dark blue color), and non-parametric bootstrap postpi (light blue color) have values of standard error improved significantly compared to the no correction (orange color). Although the correlation between y and y_p is relatively small like 0.1 - 0.2, it is still obvious that the three postpi methods give the most closed standard error values compared to the truth (grey color). Because estimates are close in different methods and centered at 0 in this example, it also leads to t-statistics centered at 0 in Figure 1(c). In Figure 1(d), we show that the three postpi methods substantially correct p-values, so the distributions of p-values of our postpi methods (green color, dark blue color, and light blue color) are very similar to the distribution of the truth (grey color), but the no correction method (orange color) has a skewed distribution.

In the second simulation example in Figure 2, we set $\beta_1 = 2$ and evaluate the performance for different methods described above. In Figure 2(a), we show that the analytical derivation postpi (green color), parametric bootstrap postpi (dark blue color), and non-parametric bootstrap postpi (light blue color) provide more accurate estimate values as the correlation between predicted and observed outcome increases. We also show that the three postpi methods significantly correct standard errors in Figure 2(b) and test statistics in Figure 2(c) compared to the no correction (orange color). The corrected standard errors and t-statistics are more close to the truth (grey color) no matter the correlation between y and y_p is small or large. In Figure 2(d), we show the distribution of $-log10$ scale of p-values. It is obvious that the distribution of no correction (orange color) is far from the truth (grey color). However, with corrections, all three postpi methods (green color, dark blue color, and light blue color) have p-value distributions closer to the truth.

B. Continuous case. In this section, we simulate continuous outcomes and covariates as described in the main text Section 2D.1.

When we evaluate the performance of different methods, we compare inference using the predicted outcome with no-correction, post-prediction inference through analytical derivation postpi, post-prediction inference through parametric bootstrap postpi, and non-parametric bootstrap postpi.

To fit the three correction approaches we first perform the following steps. In the training set, we estimate the prediction function $\hat{f}(\cdot)$. We then predict the outcome in

DRAFT

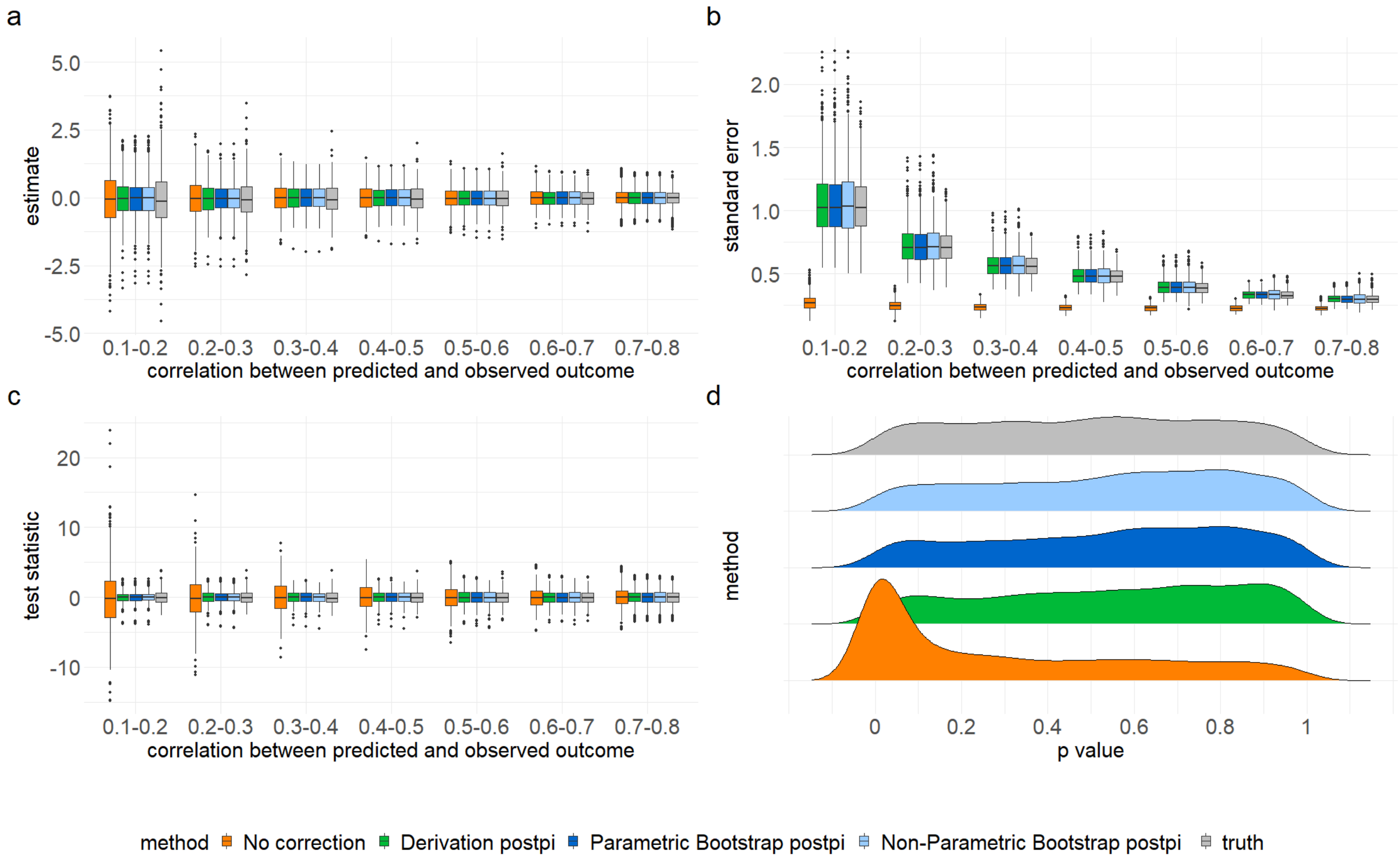

Fig. 1. Methods comparison for $\beta_1 = 0$. Data were simulated as described in Section 2A. On the x-axis for panel (a), (b), (c) are correlations between predicted and observed outcomes, and for panel (d) are p-values. On the y-axis we show (a) the estimates, (b) the standard errors, (c) the t-statistics, and (d) density distribution for different methods - no correction (orange color), analytical derivation postpi (green color), parametric bootstrap postpi (dark blue color), non-parametric bootstrap postpi (light blue color), and truth (grey color). The analytical derivation and bootstrap postpi approaches clearly improve the standard error and t-statistics values, and also correct the p-value distributions compared to no correction.

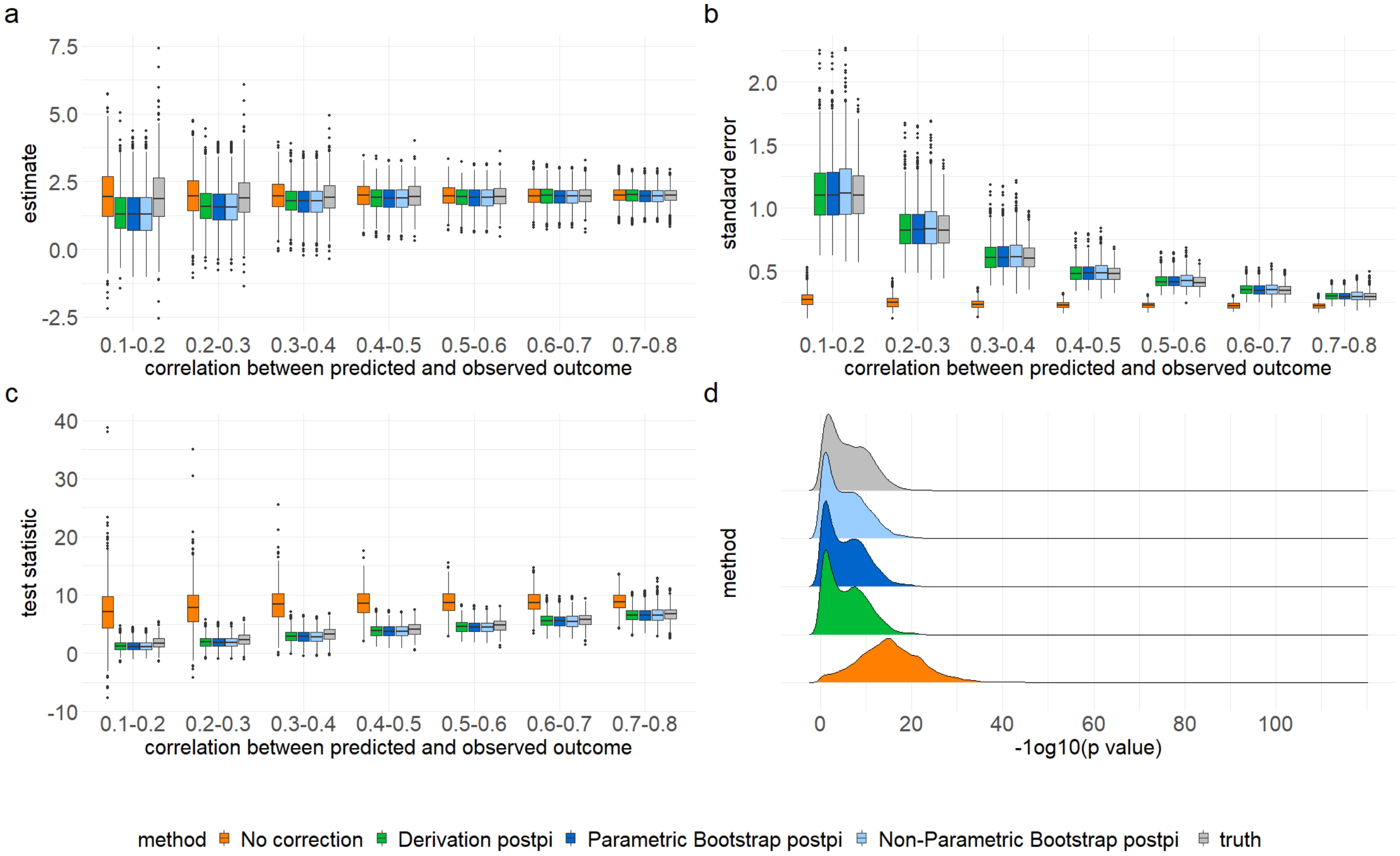

Fig. 2. Methods comparison for $\beta_1 = 2$. Data were simulated as described in Section 2A. On the x-axis for panel (a), (b), (c) are correlations between predicted and observed outcomes, and for panel (d) are p-values. On the y-axis we show (a) the estimates, (b) the standard errors, (c) the t-statistics, and (d) density distribution for different methods - no correction (orange color), analytical derivation postpi (green color), parametric bootstrap postpi (dark blue color), non-parametric bootstrap postpi (light blue color), and truth (grey color). The analytical derivation and bootstrap postpi approaches clearly improve the standard error values and correct the p-value distributions compared to no correction.

the testing and validation sets to produce outcome predictions $y_{pi} = \hat{f}(x_{i1}, x_{i2}, x_{i3}, x_{i4})$.

To fit the no correction approach, we perform a regression of the form: $E(y_{pi}|x_{i1}) = \beta_{p0} + x_{i1}\beta_{p1}$, treating the predicted outcome as if it was observed and calculate the coefficient, standard error, and t-statistic using maximum likelihood, ignoring the fact that the outcome is predicted.

To fit the postpi analytical derivation approach, we estimate the coefficient and variance using the relationship model estimated in the testing set as described in Section A.1, A.2, and A.3. We then apply these corrections to estimate the postpi analytical derivation coefficient estimator, standard error, and test statistics.

To fit the parametric and non-parametric bootstrap postpi approaches, we follow the ***Bootstrap Procedure*** Step 1-5 described in the main text Section 2B. In Step 1 we fit the GAM (2) prediction model In the training set. In Step 2 we estimate the relationship model $y_i = k(y_{pi})$ where $k(\cdot)$ is again a GAM (2) model in the testing set. In Step 3 we first set the bootstrap size $B = 100$ to start the ***for*** loop, and then repeat Step 3(i)-(v) in the validation set. Specifically, in Step 3(ii) we estimate the relationship model $k(\cdot)$ as a linear function and simulate values from the distribution: $\tilde{y}_i^b|y_{pi}^b \sim \mathcal{N}(\hat{\gamma}_0 + \hat{\gamma}_1 y_{pi}^b, \hat{\sigma}_r^2)$. Both the mean and standard deviation of the sampling distribution come from the estimated relationship model in Step 2. We then fit a linear regression model as the inference model: $E(\tilde{y}_i^b|x_{i1}^b) = \beta_{p0} + x_{i1}^b\beta_{p1}$ in Step 3(iii) and collect estimated bootstrap coefficient estimators and standard error in Step 3(iv)-(v). We finally estimate the coefficient in Step 4 and standard error in Step 5 for both the parametric and non-parametric bootstrap postpi methods.

C. Simulation example with new covariates. In this section, we show a simulation example with a new covariate introduced as the independent variable in the downstream inference model. This new covariate has not been seen in the training and testing sets, and was not used in the prediction model. However, we use this new covariate in the validation set for downstream statistical inference. We show that under such assumptions, our postpi methods do not outperform inference with no correction.

First, we simulate continuous covariates x_{ij} and error terms e_{u_i} from independent normal distributions, and simulate the observed outcome y_i using a nonlinear combination of all covariates x_{ij}. The model specification is:

$$\begin{aligned} x_{i1}, x_{i2} &\sim \mathcal{N}(1,1) \\ x_{i3} &\sim \mathcal{N}(2,1) \\ e_{u_i} &\sim \mathcal{N}(0,1) \\ y_i &= \beta_1 x_{i1} + \beta_2 x_{i2} + \beta_3 x_{i3}^2 \end{aligned} \qquad [33]$$

In each simulation cycle, we set the total sample size $n = 900$, and then create a training, testing, and validation set by randomly sampling the observed data into three equal-size groups, each with sample size 100.

In this example, we mimic a setting where the "true state of nature" model specifies that y is composed of x_{i1}, x_{i2}, x_{i3}. However, x_{i1} is unobserved in the training and testing sets, but happen to be collected in the validation set, and we are interested to infer the relationship between the outcome and x_{i1}. For the model specification, we fix the values of $\beta_1 = 2, \beta_2 = 1, \beta_3 = 1$ to generate outcomes y. In the training set, we fit a generalized additive model (GAM) (2) using all observed variables x_{i2}, x_{i3} to estimate the prediction function $y_{pi} = \hat{f}(x_{i2}, x_{i3})$. In the testing set, we apply the trained prediction model to get predicted outcomes y_{pi}. We estimate the relationship between the observed and predicted outcome (y_i and y_{pi}) as a simple linear regression model: $y_i \sim N(\gamma_0 + \gamma_1 y_{pi}, \sigma_r^2)$. Although x_{i1} is not used in the training model $\hat{f}(\cdot)$, the predicted outcomes are highly correlated with real outcomes as shown in Figure 3(a). In the validation set, we first observe that there exists a linear relationship between real outcomes y and the new covariate x_{i1} (see Figure 3(b)). However, the projection of predicted outcomes onto the new covariate x_{i1} is still essentially random (see Figure 3(c)), although the predicted outcomes are very close to real outcomes. The reason is that x_{i1} is left out of the prediction, and thus the prediction outcomes only capture signals from the included covariates – x_{i2} and x_{i3}.

For post-prediction inference, we use a linear inference model to quantify the relationship between predicted outcome y_{pi} and the new observed variable x_{i1} in the validation set. Across each simulation cycle with 100 samples, we compute the estimates, standard errors, and t-statistics of β_1 for the linear inference model using no correction (orange color) and our postpi methods (see Figure 4(a), (b), and (c)). The truth values (grey color) in Figure 4 indicates a baseline standard such that observed outcomes are used in the inference model. We conclude that our postpi methods provide the optimal correction results for post-prediction inference problems under the assumption that the training, testing, and validations sets follow the same data generating system, and the covariates used to make inference are the same covariates used in the prediction model. If this assumption no longer holds, and a new covariate is introduced in the validation set but not observed in the training and testing sets, our postpi methods do not provide the optimal solutions for post-prediction inference.

3. Applications

A. Predicting RNA quality. We consider another problem from the "Recount2" Project (https://jhubiostatistics.shinyapps.io/recount/) (3). In this example, the phenotype we care about is RNA integrity numbers (RINs). RIN is a metric to access RNA quality, a measure of how much the RNA molecules being measured have been degraded before sequencing (4). RIN has ranged from 1 to 10, with 1 being the most degraded RNA and 10 being the most intact (4). RNA-seq is a powerful technique for measuring gene expression levels in cells and tissues, but it strongly relies on the quality of input RNA (5). Because of the widespread effects of RNA quality on measurements of gene expression levels, we care about the association between RINs and gene expression levels. Studying such association is a critical step to estimate the confounding effect of RNA quality in differential expression analysis (6). Therefore, RIN is an important technological covariate in the analysis of RNA-seq (4).

Although we have gene expression level data for all "Recount2" samples, we only observe RIN values for a small subset. However, our goal is to understand which gene expressed regions are most associated with RINs in new samples (i.e. samples without observed RINs) so that we can understand which measured genes are most impacted by RNA-quality. In this example, we collected 4769 samples from the "Recount2" where we had observed RINs, as well as the predicted RINs

DRAFT

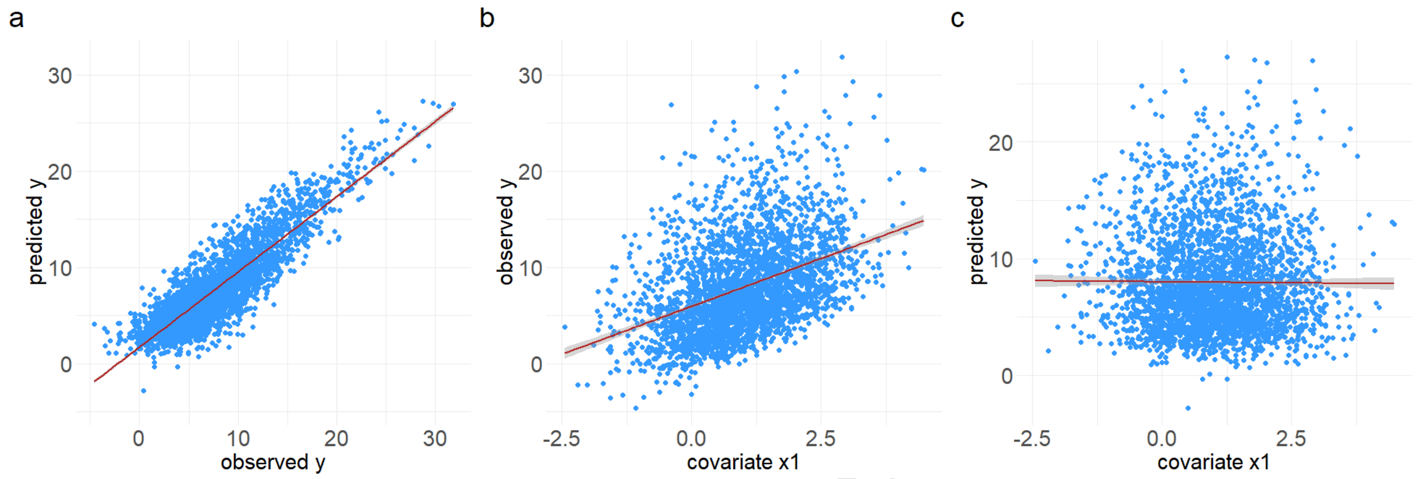

Fig. 3. Relationship scatter plot in the simulated data. Data were simulated from the ground truth model as described in Section 2C. We show (a) the relationship between the observed and predicted y, (b) the relationship between the observed y and the new covariate x_1, and (c) the relationship between the predicted y and the new covariate x_1.

DRAFT

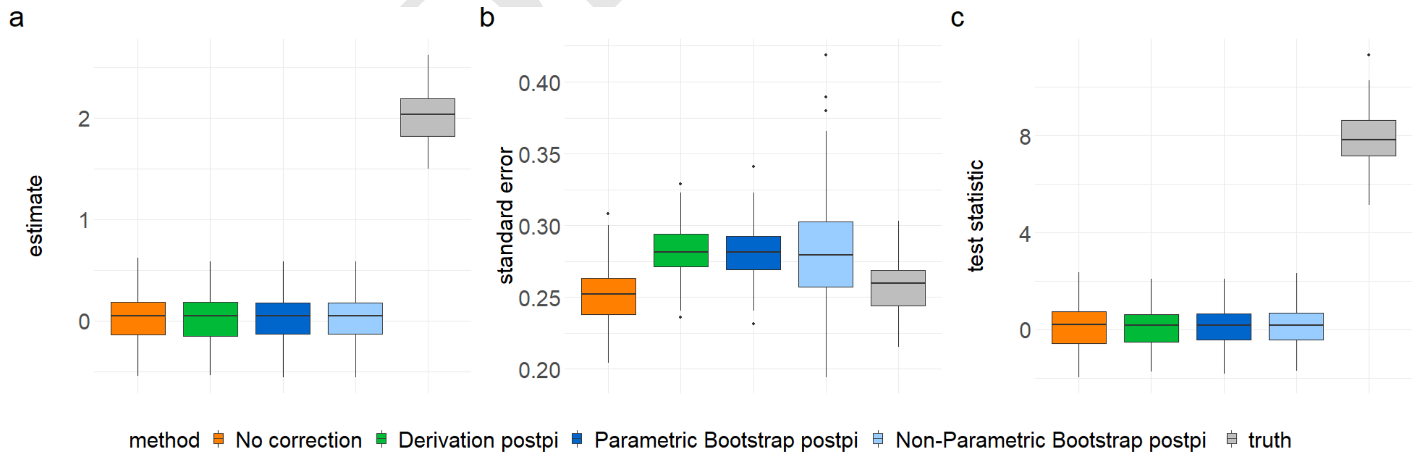

Fig. 4. Methods comparison for post-prediction inference with the new covariate. Data were simulated from the ground truth model as described in Section 2C. On the x-axis are different methods – no correction (orange color), analytical derivation postpi (green color), parametric bootstrap postpi (dark blue color), non-parametric bootstrap postpi (light blue color), and baseline (grey color). We show (a) the estimates, (b) the standard errors, and (c) the t-statistics.

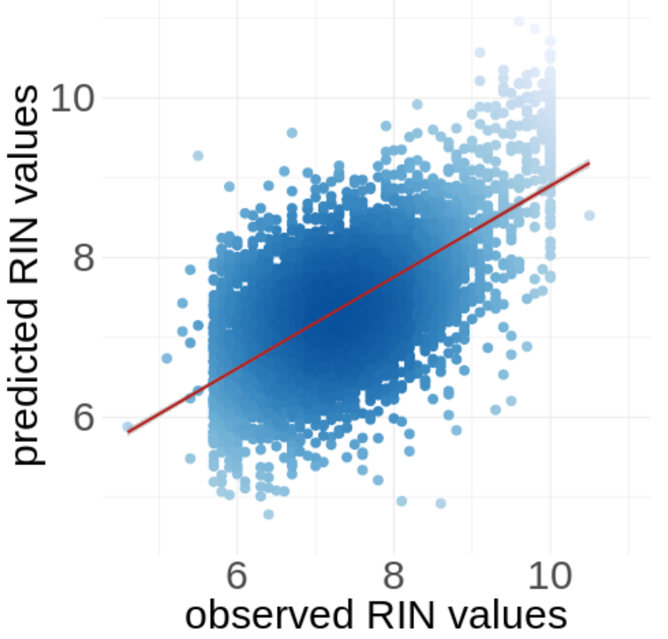

Fig. 5. Relationship between the observed and predicted RNA Quality. Data were collected from the "Recount2" Project as described in Section 3A. On the x-axis are the observed RIN values and on the y-axis are the predicted RIN values in the validation set. We observe that the observed and predicted RIN values can be modeled as a simple linear model.

which were calculated from a previously trained data set using the selected 200 expressed regions as predictors (7). In Figure 5 we show that the continuous outcomes - observed and predicted RIN values can be modeled as a simple linear relationship.

In a previous paper (7) we calculated the predicted values in a separate training set, so in this example we only separate the dataset into a testing set with a sample size $n_1 = 2383$ and a validation set with a sample size $n_2 = 2384$. The inference model we are interested in is: $E(RIN_i|ER_i^j) = \beta_0^j + \beta_1^j ER_j$. In this model, we have $j = 1, \ldots, 200$ (expressed regions) and $i = 1, \ldots, n$, n is the total number of samples in the "Recount2". Here RIN_i is the RNA quality of the ith sample, and ER_i^j is the gene expression level for the jth region on the ith sample.

In the testing set, we estimate the relationship between the observed and predicted RIN outcomes (RIN_i and RIN_{pi}) as a linear regression model. In the validation set, we again use linear regressions as the subsequent inferential models. We fit a linear regression model to each of the available expressed regions to get 200 estimates, standard errors, and t-statistics. We then compare the analytical derivation postpi, parametric bootstrap postpi, non-parametric bootstrap postpi, and the no correction approaches we did with the simulated data.

The estimates are quite similar among the three approaches in Figure 6(a) where RMSE for no correction (orange color) is 0.012 compared to the truth, 0.019 for analytical derivation postpi (green color), 0.018 for parametric (dark blue color) and non-parametric (light blue color) bootstrap postpi methods. The standard errors were underestimated by no correction (orange color) in Figure 6(b) with RMSE 0.0015, improved to 0.0014 for non-parametric bootstrap postpi (light blue color), and further reduced to 0.00008 for analytical derivation postpi (green color) and 0.00009 for parametric bootstrap postpi (dark blue color). The resulting t-statistics are in Figure 6(c) where RMSE for no correction (orange color) is 1.71 compared to the truth and improved to 1.66 for postpi bootstrap parametric (dark blue color). The analytical derivation postpi (green color) methods has RMSE 1.83 and non-parametric bootstrap postpi (light blue color) has RMSE 1.95. The analytical derivation postpi, parametric and non-parametric bootstrap postpi methods attenuate the signal when the estimates are extreme but is accurate when the estimates should be small, and all three postpi methods accurately correct the standard error of estimates. This is not surprising because we are introducing variability from the prediction model into the estimates.

1. R Core Team, *R: A Language and Environment for Statistical Computing* (R Foundation for Statistical Computing, Vienna, Austria), (2018).
2. SN Wood, Fast stable direct fitting and smoothness selection for generalized additive models. *J. Royal Stat. Soc. Ser. B (Statistical Methodol.* **70**, 495–518 (2008).
3. L Collado-Torres, et al., Reproducible rna-seq analysis using recount2. *Nat. biotechnology* **35**, 319 (2017).
4. A Schroeder, et al., The rin: an rna integrity number for assigning integrity values to rna measurements. *BMC molecular biology* **7**, 3 (2006).
5. X Adiconis, et al., Comparative analysis of rna sequencing methods for degraded or low-input samples. *Nat. methods* **10**, 623 (2013).
6. AE Jaffe, et al., qsva framework for rna quality correction in differential expression analysis. *Proc. Natl. Acad. Sci.* **114**, 7130–7135 (2017).
7. SE Ellis, L Collado-Torres, A Jaffe, JT Leek, Improving the value of public rna-seq expression data by phenotype prediction. *Nucleic acids research* **46**, e54–e54 (2018).

DRAFT

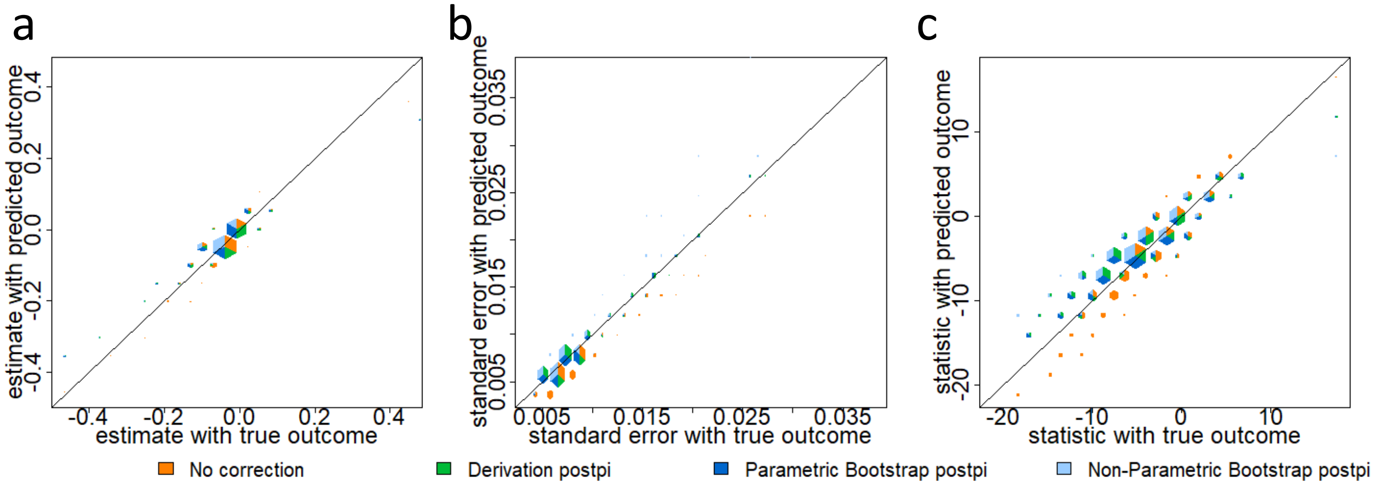

Fig. 6. RNA quality prediction. Data were collected from the "Recount2" Project as described in Section 3A. On the x-axis are the values calculated using the observed outcome and on the y-axis are the values calculated using no correction (orange color), analytical derivation postpi (green color), parametric bootstrap postpi (dark blue color), and non-parametric bootstrap postpi (light blue color). We show (a) the estimates are similar across all four approaches since the data were simulated from a normal model, (b) the standard errors are small for the uncorrected inference (orange color) but corrected with our approaches and (c) the t-statistics are anti-conservatively biased for uncorrected inference but corrected with our approaches.
